# Polydopamine–chitosan coated biofilm-state *Lacticaseibacillus paracasei* SB27 as a living band-aid for targeted colitis therapy

**DOI:** 10.1016/j.mtbio.2025.102460

**Published:** 2025-10-24

**Authors:** Yinxue Liu, Yisuo Liu, Lu Jiang, Tongjie Liu, Zhe Zhang, Pimin Gong, Huaxi Yi

**Affiliations:** College of Food Science and Engineering, Ocean University of China, Qingdao, Shandong Province 266404, China

**Keywords:** Probiotics, Biofilm, Polydopamine, Chitosan, Inflammatory bowel disease, Targeted therapy

## Abstract

Inflammatory bowel disease (IBD) involves chronic intestinal inflammation and epithelial barrier disruption. While probiotics offer therapeutic potential, planktonic (PLA) forms suffer from poor viability, limited adhesion, and suboptimal efficacy. Biofilm-state (BIO) probiotics can exert probiotic functions effectively, yet inflammation impairs the conditions necessary for probiotics to form biofilm in the intestine. Direct delivery of biofilm-state probiotics offers a more effective strategy. Here, an innovative probiotic targeted delivery system is developed by integrating biofilm-state *Lacticaseibacillus paracasei* SB27 with a rationally designed dual-coating composed of polydopamine (PDA) and chitosan (CS) (BIO@PCS). This system enables pH-responsive release of probiotics and selective adhesion to ulcerated colonic sites, mimicking a biological “band-aid”. In DSS-induced colitis model, BIO@PCS achieves superior mucosal targeting and prolonged retention compared to planktonic or uncoated forms. Upon reaching inflamed tissue, the biofilm-state *L. paracasei* SB27 rapidly forms a bacterial barrier that reinforces all four intestinal barriers and mitigates local inflammation. This approach effectively shields damaged mucosa from further injury and stabilizes the microenvironment. By enhancing both delivery efficiency and therapeutic performance, this strategy represents a dual-optimized, biofilm-based platform for IBD treatment.

## Highlights

• A dual PDA–CS coating enables targeted release and adhesion of biofilm-state probiotics to colitis sites.
• Biofilm-state *L. paracasei* SB27 shows enhanced adhesion, colonization, and immune effects over its planktonic form.
• BIO@PCS serves as a biological “band-aid” to repairing intestinal barrier integrity and rebalancing immune homeostasis.

## Graphical abstract

A biofilm-inspired probiotic delivery system is designed to alleviate colitis by targeting ulcerated intestinal regions. Encapsulation of biofilm-state *Lacticaseibacillus paracasei* SB27 within a polydopamine-chitosan coating (BIO@PCS) enhances mucosal adherence and retention. This biological “band-aid” approach effectively modulates gut microenvironments and reinforces intestinal barrier functions to mitigate inflammation.

**Figure:**
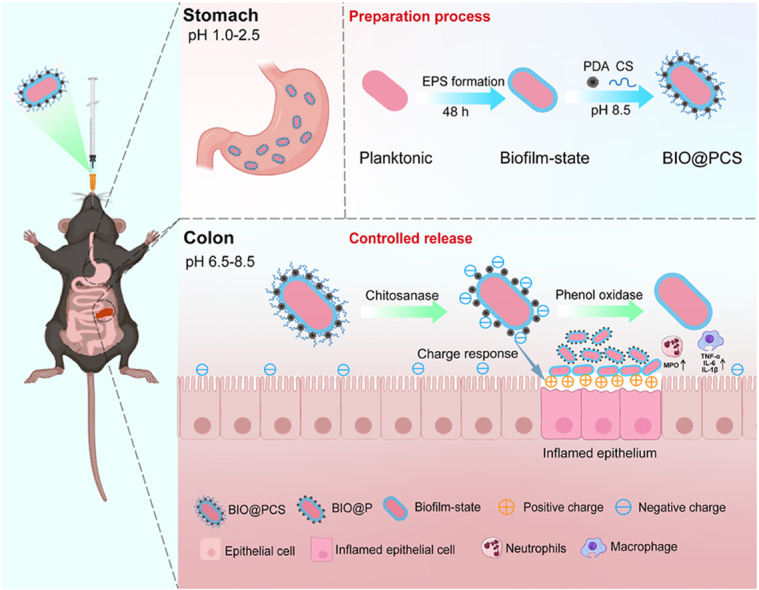

## 1 Introduction

Inflammatory bowel disease (IBD), encompassing Crohn's disease and ulcerative colitis, is a chronic intestinal disorder characterized by recurrent inflammation and mucosal injury [1]. The global IBD burden is increasing rapidly, now affecting an estimated 10 million people worldwide. China has witnessed a particularly pronounced rise in IBD prevalence, with projections suggesting 1.5 million cases by 2025 [2]. IBD pathogenesis involves the disruption of intestinal homeostasis, leading to compromised barrier function, impaired nutrient digestion and absorption, and diminished immune defense [3]. Current treatments include anti-inflammatory drugs, immunosuppressants, and biological agents, while it is often limited by suboptimal long-term safety profiles and variable patient responsiveness [4]. Probiotics are a class of microorganisms that exert health benefit to host. Probiotic-based interventions can offer dual capacity to modulate intestinal microbial ecology and promote mucosal repair.

In the intestinal environment, probiotics predominantly exist in biofilm state, which facilitates their colonization and probiotic function [5]. Bacterial biofilms are structured communities composed of bacteria and extracellular polymeric substances (EPS), which provide remarkable resistance to gastrointestinal (GI) stressors such as gastric acid and bile salts [6]. Moreover, the biofilm architecture enhances mucosal adhesion, microbial aggregation, and sustained colonization. These factors are essential for probiotics to exert effective immunomodulatory activity, reinforce the epithelial barrier, and regulate the gut microbiota [[7], [8], [9], [10], [11]]. Planktonic probiotics, by contrast, demonstrate reduced gut persistence due to susceptibility to peristaltic clearance and limited colonization capacity, and represent only a minor proportion of indigenous gut probiotics [12,13]. Currently, most probiotic supplements are administered in planktonic state. However, to persist and exert function effectively in the gut, the planktonic probiotics must transition into the biofilm state and adhere to the intestinal mucosal surface. This conversion process is particularly compromised under colitis, where mucosal injury and extracellular matrix disruption create a prohibitive microenvironment for biofilm establishment [14,15]. Therefore, these physiological limitations highlight the administration of biofilm-state probiotic supplementation as a promising therapeutic alternative to IBD. Preformed biofilm-state probiotic formulations overcome the conventional colonization barriers of planktonic probiotic, ensuring both microbial viability and sustained functionality in the gut.

In our previous study, we selected a biofilm-producing probiotic *Lacticaseibacillus paracasei* SB27 with demonstrated anti-inflammatory properties. It showed that biofilm-state *L. paracasei* SB27 exhibited enhanced adhesion and colonization capabilities *in vitro* and *in vivo* [16]. This outcome prompted us to further explore the potential of biofilm-state *L. paracasei* SB27 on the therapeutic efficacy of alleviating colitis. However, the precise localization at ulcerated sites is the essential precondition for biofilm-state *L. paracasei* SB27 to exhibit therapeutic activity. Recent advances in probiotic surface engineering have highlighted the potential of biocompatible materials to enhance probiotic therapeutic performance. Dopamine polymerization has been widely utilized as a versatile and biocompatible strategy for bacterial surface functionalization, providing strong interfacial adhesion and controllable modification [17]. Similarly, tannic acid-based metal-phenolic networks offer a robust and modular approach for engineering cell interfaces and regulating cell–microenvironment interactions [18]. Moreover, next-generation systems such as triggerable prodrug nanocoatings enable on-demand activation and co-delivery of microbial therapeutics [19], while surface topological glycosylation significantly enhances bacterial mucoadhesion and colonization [20]. Inspired by these advances, we developed an innovative biological band-aid strategy by integrating biofilm-state *L. paracasei* SB27 with an engineered dual-coating system composed of polydopamine (PDA) and chitosan (CS), aiming to achieve targeted delivery and sustained therapeutic action at ulcerated sites.

Chitosan is widely used in colon-specific drug delivery due to its rapid degradation in the colon and strong mucoadhesion to mucosal tissues [[21], [22], [23]]. Some studies have shown that chitosan-based microspheres can deliver probiotics to colonic tissues [22,24]. PDA, formed through the self-polymerization of dopamine, carries a negative charge under slightly alkaline conditions, which enable it to targetedly interact with positively charged proteins at inflamed epithelial sites [25,26]. Pan et al. demonstrated that PDA coating significantly enhanced the accumulation of modified cells in pathological tissues, which attributed to its specific affinity for inflamed colonic mucosa [27]. Therefore, the synergistic combination of chitosan and PDA created a dual-functional system. Chitosan facilitated efficient delivery and targeted release of biofilm-state probiotics to the colon, while PDA promoted selective adhesion to ulcerated tissues through pH-responsive charge interactions and enhanced localized therapeutic efficacy.

The aim of this study is to explore the targeted therapeutic efficacy of biofilm-state *L. paracasei* SB27 by an engineered dual-coating system based on PDA and chitosan. Our study might offer a novel biological band-aid strategy by forming bacterial biofilm barrier to alleviate IBD. These findings not only demonstrate the intrinsic advantages of biofilm-state probiotics in mucosal targeting and therapeutic efficacy but also provide a conceptual framework for designing next-generation probiotic delivery systems. Furthermore, this work highlights the potential of biofilms themselves as versatile biomaterials for probiotic encapsulation and targeted intervention in future clinical applications.

## 2 Materials and methods

### 2.1 Materials

*L. paracasei* SB27 was stored in the Functional Dairy and Probiotic Engineering Laboratory (Ocean University of China, Qingdao, China) and cultured in De Man, Rogosa and Sharpe (MRS) broth medium (Qingdao Hopebio Technology, Qingdao, China) at 37 °C.

Dopamine and Cy5.5-NHS were purchased from Macklin (Shanghai, China). Tris (hydroxymethyl) aminomethane hydrochloride (Tris-HCl), chitosan from shrimp shells with low viscosity, fluorescein isothiocyanate (FITC), rhodamine B, phosphate buffer saline (PBS), 2.5 % glutaraldehyde, 4 % paraformaldehyde, and total RNA extraction kit were purchased from Solarbio (Beijing, China). Dextran sulfate sodium salt (DSS) was obtained from Yeasen Biotechnology (Shanghai, China). ReverTra Ace qPCR RT Master Mix with gDNA Remover was purchased from Toyobo (Osaka, Japan). Enzyme-linked immunosorbent assay (ELISA) kits were purchased from Jonlnbio (Shanghai, China). E.Z.N.A.® soil DNA kit was purchased from Omega Bio-tek (GA, USA), and NEXTFLEX Rapid DNA-Seq kit was purchased from Bioo Scientific (TX, USA).

### 2.2 Preparation of planktonic and biofilm-state L. paracasei SB27

*L. paracasei* SB27 was cultured in MRS medium at 37 °C with shaking at 200 rpm for 24 h, then harvested by centrifugation (3000×*g*, 10 min), washed three times with PBS, and resuspended to 1 × 10^9^ CFU/mL to obtain planktonic bacteria. For biofilm formation, *L. paracasei* SB27 was inoculated into MRS medium in a 6-well plate (Costar, Corning) and incubated at 37 °C for 48 h, with a medium change at 24 h. The biofilm-state bacteria were subsequently collected by resuspension, centrifugation, and PBS washing [14].

### 2.3 Preparation of coated bacterial cells

Following the method of Pan et al. with minor modifications [27], *L. paracasei* SB27 in planktonic and biofilm-state were prepared as above described. The L. *paracasei* SB27 cells were suspended in 30 mL of 10 mM Tris-HCl buffer (pH 8.5) containing 0.3 mg/mL dopamine at a final concentration of 1 × 10^8^ CFU/mL. During the 2 h reaction, 10 μL of 3 mg/mL chitosan solution was added every 30 min. The bacterial suspension was centrifuged (3000×*g*, 10 min), resuspended in Tris-HCl buffer, and supplemented with another 10 μL of chitosan solution for an additional 30 min. After a second centrifugation, the encapsulated bacteria were resuspended in PBS, and stored at 4 °C. PLA@PCS and BIO@PCS represent planktonic (PLA) and biofilm (BIO) forms of *L. paracasei* SB27 encapsulated with PDA and chitosan. PLA@P and BIO@P were prepared solely with PDA encapsulation.

### 2.4 Characterization of coated bacterial cells

For scanning electron microscope (SEM, VEGA3 TESCAN, Czech) analysis, samples were fixed with 2.5 % glutaraldehyde, rinsed three times with PBS, dehydrated, and dried using a critical point dryer. For transmission electron microscope (TEM, JEM-1400Flash, Japan), bacterial suspensions were deposited onto a carbon-coated copper grid, washed twice with deionized water (10 min each), and air-dried. The size and zeta potential of coated bacterial cells were measured using a Zetasizer (Malvern, UK). FITC- or Rhodamine B-labeled coatings were visualized by confocal laser scanning microscope (CLSM, Nikon, Japan) and Ultraviolet–Visible (UV–Vis) absorption spectra were recorded using a UV spectrophotometer (Shimadzu, Suzhou, China). In addition, all samples were analyzed by Fourier transform infrared spectroscopy (FTIR, Nicolet iS10, Thermo Fisher Scientific, USA). Background spectra were collected prior to measurement, and transmission spectra were acquired over the range of 4000–400 cm^−1^.

### 2.5 Evaluation of the viability and tolerance of coated bacterial cells

A bacterial suspension containing 1 × 10^8^ CFUs was coated, serially diluted, and plated on MRS agar. Viable counts were determined after incubation at 37 °C for 48 h. For growth curve analysis, bacteria were inoculated into fresh medium and cultured at 37 °C; samples were collected every 2 h, and OD_600_ was measured using a microplate reader [28], with planktonic cells serving as the control. To assess tolerance, equal amount of native and coated samples were resuspended in 10 mL of medium containing bile salts (0.3 mg/mL), simulated gastric fluid (SGF, pH 3.0, with pepsin), or simulated intestinal fluid (SIF, pH 8.0, with trypsin). After incubation at 37 °C for the designated periods, 100 μL of each sample was diluted and plated on MRS agar, and CFUs were determined following 48 h incubation at 37 °C.

### 2.6 In vivo toxicity assays

SPF-grade male ICR mice were obtained from Charles River Laboratory (Beijing, China) and housed under conditions (22 ± 2 °C, 50 ± 10 % humidity, 12 h light/dark cycle). The animal protocols were approved by the Institutional Animal Care and Use Committee of the School of Food Science and Engineering at Ocean University of China (SPXY2024060720). After one-week acclimation period, ICR mice (6 weeks old, 32–34 g) were randomly divided into six groups (n = 5 per group): Control, PCS, PLA, BIO, PLA@PCS, and BIO@PCS. The control group received a daily oral gavage of 0.2 mL PBS, while the PCS group was gavaged with 0.2 mL PCS solution (0.3 mg/mL PDA + 3 mg/mL chitosan). Mice in the bacterial intervention groups were administered 0.2 mL of bacterial solution (1 × 10^9^ CFU/mL) daily. Body weight was monitored daily, and the mice were euthanized after 14 days, blood samples along with major organs were collected for further analysis.

### 2.7 Alleviating effect of coated bacterial cells on colitis

SPF-grade male C57BL/6N mice were obtained from Charles River Laboratory and the animal protocols were approved by the Institutional Animal Care and Use Committee of the School of Food Science and Engineering at Ocean University of China (SPXY2024060721). All mice (6 weeks old, 18–20 g) were randomly assigned to seven groups (n = 6 per group): Control, DSS, PCS, PLA, BIO, PLA@PCS, and BIO@PCS. The experiment lasted for 21 days. Mice in the control and DSS groups received a daily oral gavage of 0.1 mL PBS, while the PCS group received 0.1 mL PCS solution (0.3 mg/mL PDA + 3 mg/mL chitosan). The bacterial intervention groups were gavaged with 0.1 mL bacterial suspension (1 × 10^9^ CFU/mL). Mice in the control group had ad libitum access to water, while the other groups were provided free access to water for the first 14 days, followed by 7 days of 2.5 % DSS to induce colitis. Body weight was monitored daily during the modeling period, and the Disease Activity Index (DAI) was assessed according to Tong et al. [29]. On the final day of the experiment, the mice were euthanized. Colon tissues were isolated, and their lengths were measured. Serum was collected via blood centrifugation, and distal colons were fixed in 4 % paraformaldehyde for histological analysis. All other tissues were stored at −80 °C for further analysis.

### 2.8 Histopathology analysis

Colon tissue segments (0.5 cm) were fixed in 4 % paraformaldehyde and embedded in paraffin. After sectioned at 4 μm, both hematoxylin and eosin (H&E) staining and alcian blue-periodic acid-schiff (AB-PAS) staining were performed. Histological sections were evaluated for mononuclear cell infiltration, polymorphonuclear cell infiltration, epithelial hyperplasia, and epithelial injury. Each parameter was independently scored by blinded investigators on a scale of 0 (absent), 1 (mild), 2 (moderate), or 3 (severe), yielding a total score ranging from 0 to 12 [30].

### 2.9 Real-time qPCR analysis of Mucin-2 expression

The colonic tissues of mice were homogenized in TRNzol Universal reagent for total RNA extraction. Complementary DNA (cDNA) was synthesized using the ReverTra Ace qPCR RT Master Mix with gDNA Remover. Quantitative real-time PCR was performed on a CFX96 Real-Time PCR System (Thermo Fisher Scientific, USA). The expression level of the *Mucin-2* gene (forward primer: 5′-ATGCCCACCTCCTCAAAGAC; reverse primer: 5′-GTAGTTTCCGTTGGAACAGTGAA) was quantified using the 2^−ΔΔCt^ method, with β-actin (forward primer: 5′-ATCACTATTGGCAACGAGCG; reverse primer: 5′-TCAGCAATGCCTGGGTACAT) serving as the internal control.

### 2.10 ELISA assays

The serum of mice were harvested to examine the myeloperoxidase (MPO) activity and inflammatory cytokines, including IL-6, IL-1β, and TNF-α, using commercial ELISA kits. All operating procedures were carried out in strict accordance with the report of Xie et al. [28].

### 2.11 Retention of L. paracasei SB27 in the intestine

C57BL/6N mice (6 weeks old, 18–20 g) were acclimated for one week prior to the experiment, and randomly divided into four groups: PLA, BIO, PLA@PCS, and BIO@PCS. Colitis was induced with 2.5 % DSS for six days, followed by overnight fasting (water provided ad libitum). Mice were then orally gavaged with Cy5.5-NHS-labeled bacterial suspensions of *L. paracasei* SB27 (1 × 10^9^ CFU/mL) from each group. *In vivo* imaging system (IVIS) was used to monitor fluorescence signals at 6, 12, and 48 h post-gavage. After imaging, mice were euthanized, and GI tracts were excised for *ex vivo* fluorescence imaging. Fluorescence intensity was quantified using Living Image 4.4 (PerkinElmer, USA). Colon tissues were cryosectioned, DAPI-stained, and visualized by CLSM, while for TEM they were fixed, sectioned, and imaged using TEM. Additionally, GI samples were collected, and *L. paracasei* SB27 in the colon was assessed using plate counting.

### 2.12 Bacterial diversity analysis

Fecal samples after different treatments were harvested from mice and frozen at −80 °C for 16S rRNA sequencing analysis. Briefly, microbial community DNA extracted by the E.Z.N.A.® soil DNA kit, the purified PCR products were used to construct libraries using the NEXTFLEX Rapid DNA-Seq kit. The microbial community composition was subsequently determined on the Illumina MiSeq platform (Illumina, CA, USA), and all data were ultimately analyzed on the Majorbio Biocloud platform.

### 2.13 Statistical analysis

Statistical analysis was performed using GraphPad Prism 8, and data were expressed as the mean ± standard deviation. One-way analysis of variance (ANOVA) followed by Tukey's HSD test was used for the comparison of multiple groups. ∗*p* < 0.05, ∗∗*p* < 0.01, ∗∗∗*p* < 0.001 represented different statistical significances. ns stands for not significant.

## 3 Results and discussion

### 3.1 Preparation and characterization of coated probiotics

**Scheme 1:**
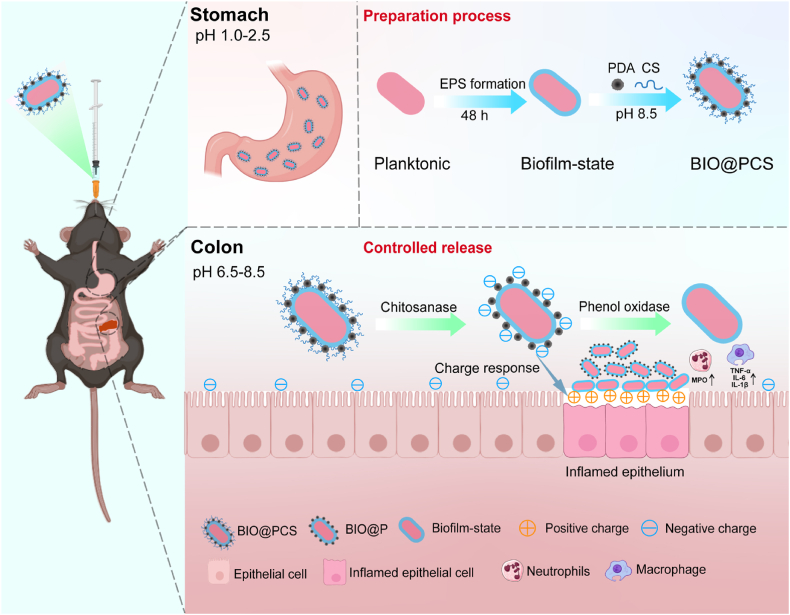
The preparation process of BIO@PCS and its mechanisms for targeted alleviation of colitis and intestinal colonization. Initially, under the protective effects of PDA, chitosan and the biofilm, BIO@PCS was successfully delivered to the colon. Upon arrival, chitosan was enzymatically degraded by gut microbiota-derived enzymes such as chitosanase and chitosan deacetylase, leading to the release of PDA-encapsulated biofilm-state *L. paracasei* SB27 (BIO@P). Subsequently, in the alkaline colonic environment, PDA-mediated charge interactions promoted the targeted adhesion of BIO@P to inflamed regions. Finally, as PDA was enzymatically degraded by phenol oxidase and amino acid deaminase, biofilm-state *L. paracasei* SB27 was fully released and exerted its functional effects at the site of inflammation.

Our previous studies showed that biofilm-state *L. paracasei* SB27 exhibited greater resilience to harsh environments, prolonged *in vivo* retention, and improved oral bioavailability compared to the planktonic form [16]. However, under disease conditions marked by altered intestinal pH, transit time, and disrupted microbiota, conventional probiotics often showed limited efficacy due to their non-specific distribution in the gut [31,32]. To enhance targeted delivery to intestinal lesions, we integrated chitosan and PDA into the biofilm matrix. Chitosan, a naturally cationic polysaccharide, has been extensively used as a carrier for colon-targeted drug delivery due to its susceptibility to enzymatic degradation by gut microbiota-derived enzymes, including chitosanase and chitosan deacetylase [22]. Beyond this degradability, chitosan promotes probiotic adhesion by electrostatically interacting with negatively charged epithelial surfaces and mucus, thereby enhancing initial attachment and extending intestinal retention [19]. Furthermore, the degradation products of chitosan, such as chitosan oligosaccharides and low molecular weight chitosan, can transiently form an adhesive network on the inflamed mucosal surface, providing additional support for probiotic protection and promoting their colonization in the inflamed gut environment [23]. PDA, formed through the oxidative self-polymerization of dopamine under mildly alkaline conditions (pH 8.5), contained catechol, phenolic hydroxyl, amide, and amine groups that provided strong adhesive properties [17,25,33]. These functional groups enabled PDA to bond with amino, hydroxyl, and carboxyl groups on chitosan or bacterial surfaces through hydrogen bonds, covalent Michael addition, and Schiff base reactions. Simultaneously, chitosan electrostatically adsorbed onto probiotics surfaces, and stabilized the probiotics-PDA-chitosan composite structure (Scheme 1) [26,34].

**Fig. 1:**
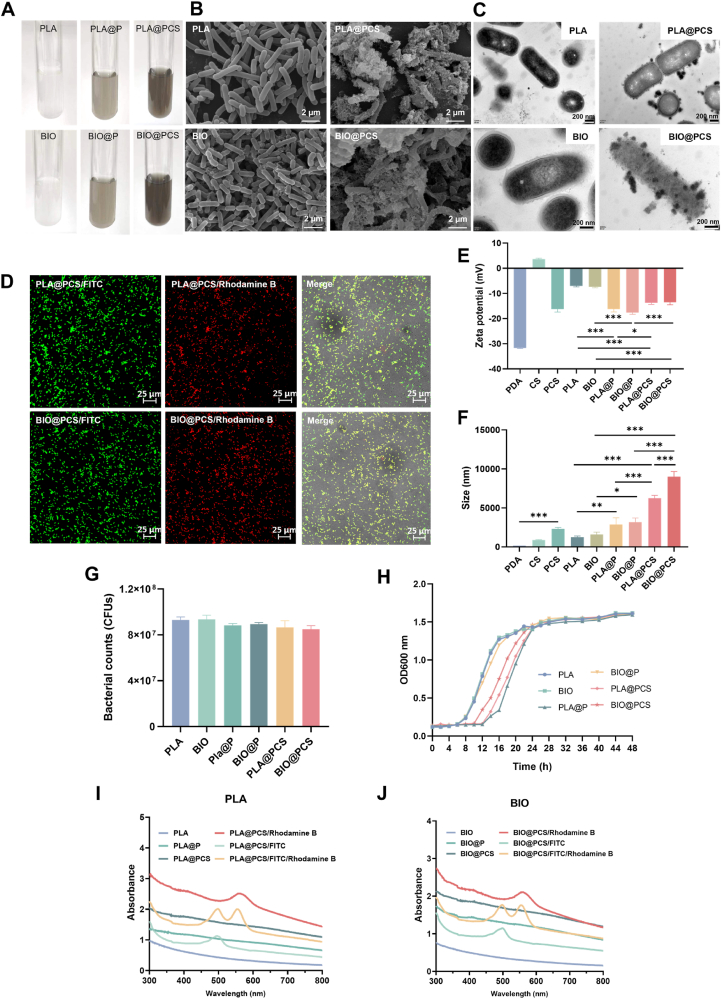
Preparation and characterization of coated bacteria. **A**) Digital photos of equal amount of native and coated *L. paracasei* SB27 suspended in PBS. **B**) Typical SEM images of PLA, PLA@PCS, BIO and BIO@PCS. **C**) Typical TEM images of PLA, PLA@PCS, BIO and BIO@PCS. **D**) Confocal images of PLA@PCS and BIO@PCS. **E**) Zeta potentials of native and coated *L. paracasei* SB27. **F**) Size of native and coated *L. paracasei* SB27. **G**) Bacterial viability of native and coated *L. paracasei* SB27. **H**) Growth curves of native and coated *L. paracasei* SB27. **I**) UV–Vis absorption spectra of native and coated planktonic *L. paracasei* SB27. **J**) UV–Vis absorption spectra of native and coated biofilm state *L. paracasei* SB27. All fluorescence images were acquired with consistent exposure and laser intensity settings, and consistently processed (brightness/contrast adjustment and background subtraction) for comparability. Data are presented as means ± SD (n = 3). Significance was assessed by one-way analysis of variance (ANOVA) with Tukey post hoc test, giving *p*-values, ∗*p* < 0.05, ∗∗*p* < 0.01, ∗∗∗*p* < 0.001.

As shown in Fig. 1A, suspensions of both planktonic and biofilm-state *L. paracasei* SB27 exhibited a gray color when coated with PDA alone, while co-deposition with chitosan and PDA resulted in a darker appearance. SEM and TEM revealed substantial deposition of PCS nanoparticles on the surfaces of both planktonic and biofilm-state *L. paracasei* SB27 cells, forming a tight encapsulation around the bacteria. (Fig. 1B–C). Notably, biofilm-state *L. paracasei* SB27 demonstrated significantly greater adsorption of PCS nanoparticles than the planktonic counterpart, likely due to its complex EPS structure, abundant surface functional groups, and enhanced adhesive properties [6,13]. CLSM further validated the successful co-deposition of chitosan and PDA. FITC-labeled chitosan and Rhodamine B-labeled PDA produced overlapping green and red fluorescence in both PLA@PCS and BIO@PCS, confirming the integrated coating (Fig. 1D). Additionally, zeta potential measurements showed a significant reduction in the biofilm-state of *L. paracasei* SB27 after coating with PDA and PCS, with values decreasing from −7.36 mV to −17.60 mV and −13.53 mV, respectively, indicating the incorporation of both positively charged chitosan and negatively charged PDA (Fig. 1E). The size of BIO@PCS increased from 1608.33 nm to 9020.67 nm, and 2762.67 nm thicker than that of PLA@PCS, likely due to the inherent thickness of the biofilm layer and its enhanced affinity for PCS coating (Fig. 1F). Bacterial viability and growth were assessed by colony counting and OD_600_. Encapsulated L. *paracasei* SB27 maintained 90 % survival, and the growth kinetics over 24 h showed negligible impact of the coating, confirming that encapsulation preserved bacterial activity and proliferative capacity (Fig. 1G–H). Additionally, the UV–Vis spectra of PLA@PCS and BIO@PCS displayed characteristic absorption peaks at 556 nm and 492 nm, corresponding to Rhodamine B and FITC, respectively (Fig. 1I–J). FTIR analysis of PDA and CS coatings (Fig. S1) revealed consistent patterns in both planktonic and biofilm groups. Relative to uncoated PLA and BIO, PLA@P, BIO@P, PLA@PCS and BIO@PCS showed a red-shift and broadening of the O–H/N–H band (3700–3000 cm^−1^), indicating reinforced hydrogen bonding between coating moieties and bacterial surfaces [35]. In the amide region (1700–1480 cm^−1^), PLA@P and BIO@P exhibited an amide I shift from 1700 to 1620 cm^−1^ and a new amide II peak at 1540 cm^−1^, consistent with PDA aromatic C=C and C–N vibrations, confirming interactions between surface proteins and polysaccharides. PLA@PCS and BIO@PCS also displayed amide I at 1620 cm^−1^ but with a stronger amide II at 1490 cm^−1^, reflecting overlapping PDA and chitosan signals [36]. Importantly, PLA@PCS and BIO@PCS presented intensified polysaccharide fingerprints at 1160 and 1070 cm^−1^, directly attributable to chitosan glycosidic and hydroxyl vibrations [37]. Together, these spectral shifts provided direct evidence for the successful deposition of a compact PDA–CS composite encapsulation layer on bacterial surfaces, which was in agreement with previously reported results [27].

To systematically evaluate the protective effects of PDA and chitosan coatings on *L. paracasei* SB27 under physiological stress, we assessed their tolerance to bile salts, simulated gastric fluid, and simulated intestinal fluid. In 0.3 % bile salt MRS medium, uncoated planktonic cells showed a sharp viability decline from 5.4 × 10^8^ to 2.1 × 10^8^ CFU/mL within 3 h, whereas PDA- and PDA–CS-coated cells (PLA@P, PLA@PCS) maintained significantly higher counts. Notably, coated biofilm cells (BIO@P, BIO@PCS) exhibited increased viability after 3 h, indicating synergistic protection from both coating and biofilm barriers (Fig. S2A). In simulated gastric fluid, BIO@P, PLA@PCS, and BIO@PCS retained almost unchanged viability over 3 h, demonstrating effective acid protection (Fig. S2B). In simulated intestinal fluid, uncoated cells showed a viability drop after 5 h, while BIO, BIO@P, PLA@PCS, and BIO@PCS maintained or increased counts, confirming enhanced stability and survival in the intestinal environment (Fig. S2C). Overall, PDA–CS encapsulation markedly enhanced the survival of *L. paracasei* SB27 under bile salt and GI conditions, with a more pronounced effect on biofilm cells. Moreover, the combined action of PDA and chitosan outperformed PDA alone, consistent with the findings reported by Pan et al. [27].

### 3.2 BIO@PCS exhibited excellent *in vivo* biosafety

**Fig. 2:**
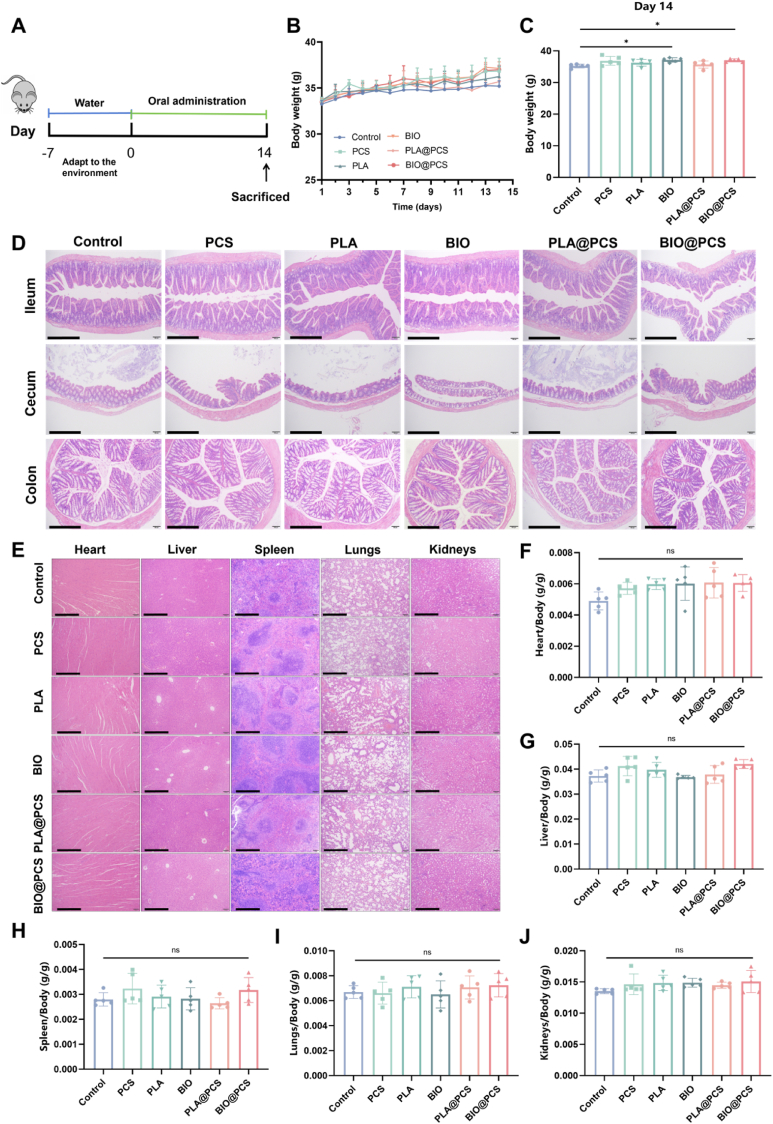
*In vivo* toxicity analysis. **A**) The experimental design for *in vivo* safety evaluation. **B**) Changes in body weight during the 14-day experimental period. **C**) Change in body weight on the 14th day of the experiment. **D**) Typical images of H&E staining of the ileum, cecum and colon. Scale bars: 400 μm. **E**) Typical images of H&E staining of the heart, liver, spleen, lungs and kidneys. Scale bars: 400 μm. **F - J**) Organ index of heart **F**), liver **G**), spleen **H**), lungs **I**) and kidneys **J**). Data are presented as means ± SD (n = 5). Significance was assessed by one-way analysis of variance (ANOVA) with Tukey post hoc test, giving *p*-values, ∗*p* < 0.05, ∗∗*p* < 0.01, ∗∗∗*p* < 0.001; ns, not significant.

A comprehensive *in vivo* safety evaluation of BIO@PCS was conducted, which serves as a critical prerequisite for its functional application. This assessment aimed to validate its biocompatibility and suitability for clinical translation, and the experimental process was summarized in Fig. 2A. The results showed that no mortality or adverse health effects were observed in the mice. Body weight measurements showed no significant differences between groups before treatment. After administration, all groups gained weight, and the BIO and BIO@PCS groups exhibited notably greater weight gain by Day 14, suggesting that biofilm-state *L. paracasei* SB27 may promote growth and development in mice (Fig. 2B–C). The histological examinations of major visceral organs and intestines were carried out, including the heart, liver, spleen, lungs, kidneys, ileum, cecum, and colon. H&E staining revealed normal histological structures in all internal organs and intestines of all groups (Fig. 2D–E). When foreign substances invade the body, they may cause either organ atrophy or enlargement, which can be effectively assessed using organ indices [38]. The results indicated no significant differences in organ indices between the experimental groups and the control group (Fig. 2F–J). Furthermore, hematological analysis revealed that the levels of inflammatory cytokines in the experimental groups were comparable to those in the control group, further confirming the biocompatibility of BIO@PCS (Fig. S3A–D). These findings underscored the safety and clinical translational potential of BIO@PCS. Subsequent investigations focused on validating the sustained therapeutic effects of BIO@PCS in colitis models.

### 3.3 BIO@PCS significantly ameliorated DSS-induced colitis in mice

**Fig. 3:**
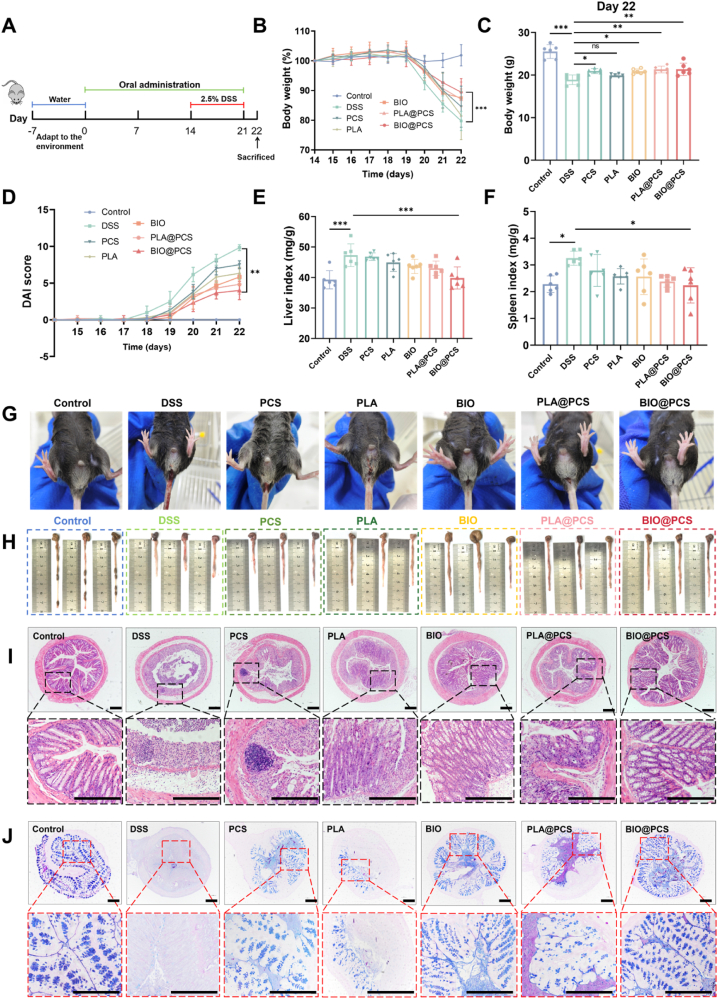
Alleviating effect of BIO@PCS in a DSS-induced murine model of colitis. **A**) The experimental design for colitis treatment. **B**) Changes in body weight during the 22-day experimental period. **C**) Change in body weight on the 22nd day of the experiment. **D**) Disease activity index (DAI) variation of mice among different groups as a function of time. **E**) The changes in liver index in different groups. **F**) The changes in spleen index in different groups. **G**) Representative digital photos of rectal areas in different groups. **H**) Digital photos of cecum-colon tissues. **I**) Representative images of H&E staining of colon tissues harvested on day 22 from different groups. Scale bars: 200 μm. **J**) AB-PAS staining of colon tissues from different groups. Scale bars: 200 μm. Data are presented as means ± SD (n = 6). Significance was assessed by one-way analysis of variance (ANOVA) with Tukey post hoc test, giving *p*-values, ∗*p* < 0.05, ∗∗*p* < 0.01, ∗∗∗*p* < 0.001; ns, not significant.

Based on the excellent biosafety of BIO@PCS, we evaluated its therapeutic potential in alleviating ulcerative colitis in mice. The experimental procedure was summarized in Fig. 3A. The therapeutic efficacy was firstly evaluated by measuring changes in body weight, a key parameter for monitoring colitis progression (Fig. 3B). Colitis mice experienced significant body weight loss from day 19, and the body weight of the DSS model group decreased by 20.23 % on day 22 compared to day 1, while those treated with PCS, PLA, BIO, PLA@PCS, and BIO@PCS showed comparatively less weight loss at 15.44 %, 18.43 %, 12.46 %, 12.94 %, and 10.56 %, respectively (Fig. 3C). Compared to planktonic *L. paracasei* SB27, the biofilm-state strain exhibited a more pronounced effect in alleviating body weight loss in mice. Furthermore, DSS-induced mice exhibited symptoms including diarrhea, bloody stools, increased DAI scores, and elevated liver and spleen indices. Treatment with BIO@PCS and biofilm-state *L. paracasei* SB27 significantly alleviated these symptoms and reduced DAI scores and organ indices (Fig. 3D–G).

The ameliorative efficacy was further assessed through histopathological analysis. Colon shortening and altered gross morphology are hallmark features of DSS-induced colitis [39]. This condition typically leads to significant pathological changes, including the loss of goblet and epithelial cells, crypt destruction, and inflammatory cell infiltration. Notably, treatments with BIO@PCS and BIO significantly alleviated colon shortening and improved histological architecture compared to other groups (Fig. 3H–I, S4, S5). Tight junction proteins are the primary connections between intestinal epithelial cells and are essential for maintaining the mechanical integrity and normal function of the intestinal mucosal barrier [40]. Given that tissue damage in IBD is often accompanied by increased intestinal permeability, alterations in the expression of tight junction proteins, such as Occludin, may contribute to this phenomenon [41]. Immunofluorescence staining demonstrated that DSS exposure markedly decreased the expression of Occludin protein in colon tissues, whereas intervention with biofilm-state *L. paracasei* SB27 effectively preserved its high expression level (Fig. S6A–B). These findings suggested that, compared to the planktonic *L. paracasei* SB27, the biofilm-state strain offered more effective protection against damage to the intestinal mechanical barrier in mice.

AB-PAS staining revealed that DSS-induced colitis caused a marked disruption of the colonic mucus layer, characterized by a near-complete depletion of light blue-stained mucus and a substantial loss of goblet cells (Fig. 3J–S7). The BIO and BIO@PCS groups preserved the structural integrity of the mucus layer and maintained goblet cell morphology similar to that of the healthy control group, demonstrating the strongest protective effects of biofilm-state *L. paracasei* SB27. Moreover, Mucin-2, a highly glycosylated protein secreted by goblet cells and a major structural component of the intestinal mucus layer, was significantly upregulated in the colonic tissue of mice following treatment with biofilm-state *L. paracasei* SB27 (Fig. S8). The upregulation of Mucin-2 enhanced the chemical barrier of the intestine, and strengthened the resistance to pathogenic invasion and exogenous insults, thereby improving the lubricative and protective functions of the intestinal mucus layer. Mechanistically, this effect may arise from enhanced probiotic–host interactions within the biofilm microenvironment of *L. paracasei* SB27. In the biofilm state, bacteria released structural components and metabolites, such as butyrate, which acted as a histone deacetylase inhibitor, to stimulate Mucin-2 transcription and secretion in goblet cells [12,42]. Additionally, Yao et al. found that microbial pattern recognition induced the expression of sialyltransferase ST6GALNAC1 in goblet cells, which promoted the sialylation of Mucin-2 and enhanced its resistance to bacterial proteolytic degradation, thereby preserving mucus integrity [43]. *L. paracasei* also strengthened the intestinal barrier by inhibiting the NF-κB/MLCK pathway and upregulating tight junction proteins (e.g., ZO-1, ZO-2), further supporting Mucin-2 expression [44]. Together, these pathways suggest that biofilm formation not only enhances colonization and persistence of *L. paracasei* SB27, but also promotes Mucin-2 synthesis and stabilization, thereby reinforcing the mucus barrier and facilitating mucosal healing.

To further elucidate the underlying mechanism of mitigating intestinal inflammation by BIO@PCS, we quantified key inflammatory cytokines in colonic tissues using ELISA kits. Notably, treatment with BIO@PCS and BIO resulted in a marked reduction in pro-inflammatory cytokines, including IL-1β, IL-6, and TNF-α, highlighting the robust anti-inflammatory properties of biofilm-state *L. paracasei* SB27 (Fig. S9A–C). Additionally, the activity of MPO, a hallmark of neutrophil infiltration, was significantly decreased following both BIO@PCS and BIO treatments (Fig. S9D). The improved treatment efficacy could be explained by the enhanced survival and accumulation of biofilm-state *L. paracasei* SB27 in local inflamed tissue, which was able to suppress the expressions of proinflammatory cytokines and chemokines [45,46]. Collectively, these findings indicated that biofilm-state *L. paracasei* SB27 possessed a superior ability to protect the immune barrier of inflamed colonic tissue compared to its planktonic counterpart.

### 3.4 Enhanced survival and targeted mucoadhesion of BIO@PCS

**Fig. 4:**
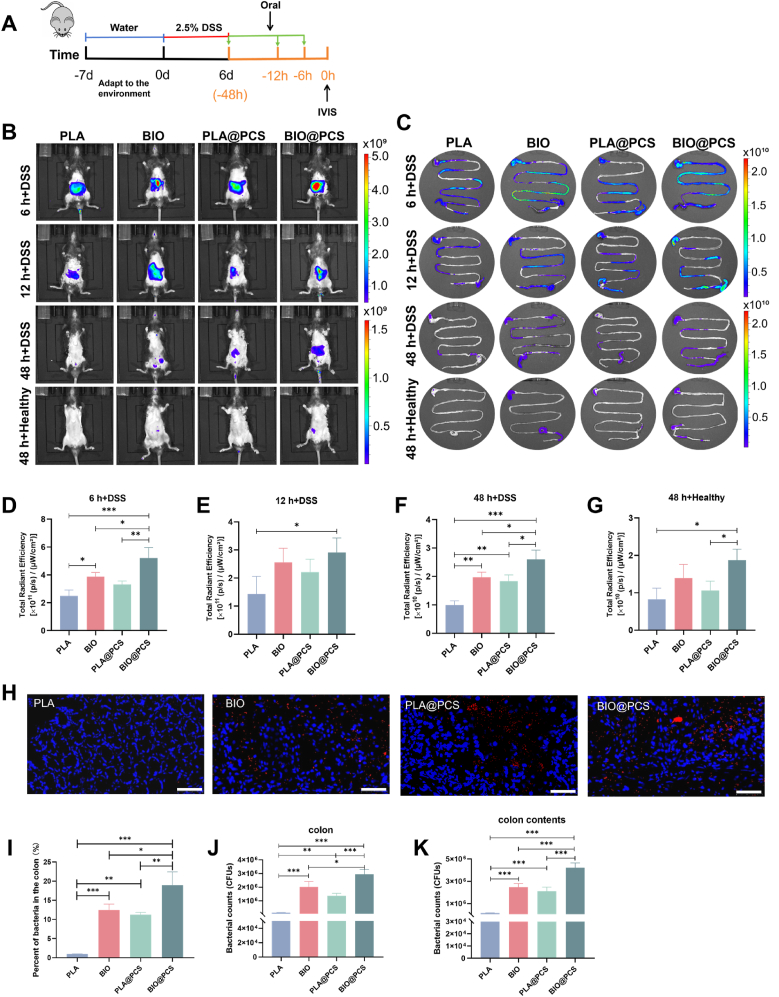
The targeting effect of BIO@PCS in the inflamed colon of mice. **A)** The experimental design for targeting effect. **B)** Fluorescence images of healthy and DSS mice at different times after oral administration of PLA, BIO, PLA@PCS and BIO@PCS. **C**) Fluorescence images of the mouse GI tract at different times after oral administration of PLA, BIO, PLA@PCS and BIO@PCS. **D - F)** Fluorescence intensity in the GI tract of DSS-induced mice at 6 h **D)**, 12 h **E)**, and 48 h **F)** post-gavage. **G)** Fluorescence intensity in the GI tract of healthy mice at 48 h post-gavage. **H)** Typical fluorescent slices of colon sampled from DSS-induced mice. The blue channel refers to 4′,6-diamidino-2-phenylindole (DAPI) and red channel indicates Cy5.5-NHS-labeled *L. paracasei* SB27. Scale bars: 50 μm. **I)** Percentage accumulation of *L. paracasei* SB27 in the inflamed colon 12 h post-administration. **J - K**) Bacterial retention in the colon **J**) and colonic contents **K**) at 12 h post-gavage. Tissues were sampled and homogenized for bacterial plate counting. All fluorescence images were acquired with consistent exposure and laser intensity settings, and consistently processed (brightness/contrast adjustment and background subtraction) for comparability. The color scale was optimized to clearly visualize biodistribution differences across time points: higher ranges were used for the 6 h + DSS and 12 h + DSS groups, and lower ranges for the 48 h + DSS and 48 h + Healthy groups. Data are presented as means ± SD (n = 3). Significance was evaluated by one-way (ANOVA) with Tukey post hoc test, giving *p*-values, ∗*p* < 0.05, ∗∗*p* < 0.01, ∗∗∗*p* < 0.001. (For interpretation of the references to color in this figure legend, the reader is referred to the Web version of this article.)

Targeted oral delivery of probiotics to inflamed colonic regions remains challenging due to disruption of the mucosal barrier, fluctuating intestinal pH, altered transit times, and dysbiosis associated with disease states [47]. Severe mucosal injury further compromised probiotic adhesion and colonization [48]. To address this, we evaluated the biodistribution and retention of *L. paracasei* SB27 in mice using IVIS imaging (Fig. 4A). The fluorescence signal of planktonic *L. paracasei* SB27 in the abdominal region declined sharply within 12 h post-administration, whereas the BIO@PCS formulation sustained detectable signals up to 48 h (Fig. 4B). Consistent with these observations, *ex vivo* imaging of intestinal tissues further confirmed enhanced probiotic retention. At 12 h, the fluorescence intensity throughout the GI tract in the BIO@PCS, PLA@PCS, and BIO groups was approximately 2.0-, 1.5-, and 1.8-fold higher than that in the PLA group respectively (Fig. 4C–F). A comparable pattern was observed in the colon, where the BIO@PCS, PLA@PCS, and BIO groups exhibited 4.5-, 2.6-, and 2.8-fold stronger signals, respectively (Fig. S10A–D). Collectively, across all time points, BIO@PCS displayed the strongest retention, followed by BIO, PLA@PCS, and PLA, indicating the synergistic effect of biofilm formation and PCS coating. Notably, at 48 h post-gavage, the probiotic retention was markedly enhanced in colitis mice compared with healthy controls, particularly in the BIO@PCS group, as evidenced by elevated fluorescence in the GI tract (Fig. 4F–G), and colon (Fig. S10D–E). This outcome was anticipated, as the polyphenol-based complex carries a net negative charge that enables BIO@PCS to bind electrostatically to cation-rich inflammatory sites, thereby enhancing colonization. Similar findings were reported by Zhu et al. [49], who designed a nanocoating composed of procyanidin, Fe(III), and high-molecular-weight hyaluronan (HMW-HA) to rapidly modify the surface of *E. coli Nissle 1917* (EcN). Using IVIS imaging, they observed significantly higher fluorescence intensity of EcN-mCherry@PC-Fe/HA in inflamed colons compared with healthy controls, confirming improved adhesion and retention at inflammatory sites. Consistently, Huang et al. [50] demonstrated that hydrogel-encapsulated *Lactobacillus reuteri* (HA-LR) exhibited enhanced targeting of inflamed colonic mucosa, as indicated by increased radiant efficiency and higher probiotic counts in colitic mice after oral administration. Together, these findings parallel our observations and underscore that the negative surface charge of encapsulating materials facilitates binding to positively charged inflamed epithelial cells, thereby prolonging probiotic retention at diseased sites.

To further quantify colonic colonization, viable plate counting was performed. At 48 h post-administration, BIO@PCS, PLA@PCS, and BIO groups exhibited 23.2-, 10.7-, and 16.0-fold higher survival in the colon compared with planktonic bacteria respectively. A similar pattern was observed in colonic contents, where BIO@PCS, PLA@PCS, and BIO groups showed 27.8-, 13.8-, and 16.2-fold greater viability, respectively (Fig. 4J–K). Consistent with these findings, the fluorescence imaging of colonic tissues revealed a pronounced increase in *L. paracasei* SB27 colonization upon biofilm and PCS coating, with targeted colonization rates rising from 1 % to 19 % (Fig. 4H–I). TEM observation provided further direct evidence of the targeted colonization of BIO@PCS in the inflamed colon (Fig. S11A–C). The markedly enhanced colonization in biofilm-state probiotics can be attributed to the multifaceted functionality of EPS. Beyond serving as a physical barrier, the EPS matrix actively promotes mucosal adhesion through a combination of non-specific physicochemical interactions, such as electrostatic and hydrophobic forces with mucosal components and potential specific molecular recognition between bacterial adhesions and host receptors [[9], [10], [11]]. This robust interfacial interaction, augmented by the physical entrapment of the biofilm within the mucus network, underlies the superior colonization capability of biofilm-state *L. paracasei* SB27 over their planktonic counterparts. Collectively, these results demonstrate that BIO@PCS not only improved *L. paracasei* SB27 survival and persistence within the intestine but also achieved targeted adhesion to inflamed colonic regions, supporting the concept that biofilm-state probiotics could act as a biological band-aid for localized therapy.

Notably, it was observed that BIO@PCS formulation exhibited superior targeting and prolonged retention. However, the BIO group alone also showed markedly enhanced performance. In particular, biofilm-state *L. paracasei* SB27 significantly outperformed the planktonic group and demonstrated slightly better efficacy than PLA@PCS. Together, these results underscore the intrinsic advantages of the biofilm phenotype in promoting colonization and persistence. To our knowledge, this study provides the first evidence that the biofilm phenotype enhances the targeted adhesion capacity of probiotic strains within the GI tract. We hypothesize that this effect arises from the unique structural and functional attributes of the biofilm. The EPS within the biofilm matrix, enriched in polysaccharides and proteins, likely mediate the specific recognition and interactions of probiotics with epithelial cells or extracellular matrix components at inflamed sites [51,52]. In addition, quorum sensing within the biofilm community may further promote coordinated adhesion and colonization by regulating collective bacterial behaviors through the secretion and detection of signaling molecules, such as autoinducing peptides or acyl-homoserine lactones [13,15]. Moreover, biofilm-state bacteria may counteract elevated levels of reactive oxygen species (ROS) characteristic of inflamed tissues by secreting antioxidant enzymes such as catalase, thereby enhancing both their survival and therapeutic potential [53,54].

In addition to enhanced targeting and adhesion, the prolonged retention and sustained efficacy of BIO@PCS can be attributed to its controlled release, governed by the sequential enzymatic degradation of the PDA–CS coating. The dual-coating system operates in a stepwise manner: the chitosan coating initially acts as a protective shuttle for targeted colonic delivery and adhesion. Its gradual enzymatic degradation by gut microbiota-derived enzymes (e.g., chitosanase and chitosan deacetylase) [22,55] subsequently triggers the release of PDA-encapsulated biofilm-state *L. paracasei* SB27 (BIO@P). In the mildly alkaline colonic environment, the negatively charged PDA coating then facilitates selective adhesion of BIO@P to inflamed epithelial tissues through charge interactions, ensuring prolonged residence at the lesion site. The subsequent enzymatic degradation of PDA by phenol oxidase and amino acid deaminase [17,25,27] enables the progressive release and reactivation of biofilm-state bacteria, thereby maintaining continuous probiotic colonization and biofilm formation. This sustained-release process acts as a biological band-aid, providing persistent mucosal protection and promoting long-term restoration of intestinal homeostasis.

Collectively, these findings highlight the distinct yet complementary functions of chitosan and PDA within the dual-coating system. Chitosan provides mucoadhesion, biodegradability, and colon-specific protection that facilitate initial delivery and adhesion, whereas PDA provides robust interfacial binding and pH-responsive targeting to inflamed mucosa. The deficiency of either component resulted in diminished stability, targeting accurary, or retention, thereby underscoring their synergistic contribution to therapeutic efficacy.

### 3.5 Regulation of the gut microbiota by BIO@PCS in colitic mice

**Fig. 5:**
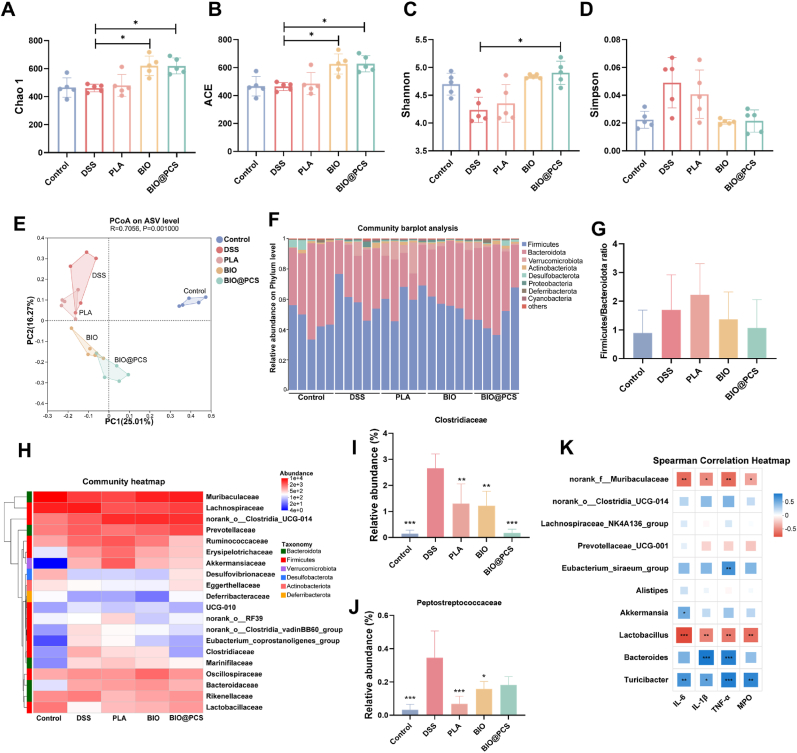
Modulation of gut bacteria by planktonic and biofilm-state *L. paracasei* SB27 and BIO@PCS during colitis treatment. Comparison of alpha diversity assessed by **A)** Chao1 index, **B)** ACE index, **C)** Shannon index, and **D)** Simpson index. **E)** Clustering of gut microbial communities for different experimental groups based on the PCoA plot with Weighted UniFrac distance. **F)** Histogram of the relative abundances of different species on phylum level. **G)** The ratio of Firmicutes to Bacteroides. **H)** Clustering heatmap of species abundance at family level. **I)** Relative abundance of Clostridiaceae. **J)** Relative abundance of Peptostreptococcaceae. **K)** Association heatmap of gut microbiota and pro-inflammatory factors in colon of mice. Data are presented as means ± SD (n = 5). Significance was assessed by one-way analysis of variance (ANOVA) with Tukey post hoc test, giving *p*-values, ∗*p* < 0.05, ∗∗*p* < 0.01, ∗∗∗*p* < 0.001.

The intestinal microbiota plays a central role in maintaining immune homeostasis, and its disruption is one of drivers of IBD pathogenesis [56]. To explore whether BIO@PCS could counteract DSS-induced dysbiosis and restore microbial balance, we performed high-throughput 16S rRNA sequencing of fecal samples. Microbial diversity was assessed using Chao1, ACE, Shannon, and Simpson indices, which served as key indicators of species richness and community diversity. DSS administration significantly reduced both richness and diversity, which was consistent with previous reports of microbial collapse during colitis [57,58]. In contrast, BIO@PCS treatment demonstrated a significant reversal of this decline, restoring diversity to levels comparable with healthy controls (Fig. 5A–D). A higher Shannon index and lower Simpson index were characteristic of a more balanced microbial community [59], suggesting that BIO@PCS preserved microbial ecosystem stability under inflammatory stress. Principal coordinate analysis (PCoA) further revealed that BIO and BIO@PCS reshaped the overall microbial composition toward that of healthy mice, effectively counteracting the DSS-induced deviation (Fig. 5E). Together, these results demonstrated that biofilm-state *L. paracasei* SB27 protected against the loss of richness and diversity imbalance characteristic of colitis.

Taxonomic analysis provided mechanistic insight into these changes. At the phylum level, Bacteroidetes and Firmicutes remained dominant (Fig. 5F). DSS treatment increased the Firmicutes/Bacteroidetes (F/B) ratio, a dysbiosis marker linked to intestinal inflammation [60], whereas BIO and BIO@PCS significantly reduced this ratio (Fig. 5G). Moreover, all probiotic groups suppressed Proteobacteria expansion (Fig. S12A), a phylum enriched in pathogenic bacteria and frequently regarded as a signature of colitis [61]. At the genus level, BIO@PCS promoted enrichment of beneficial taxa such as *Lactobacillus* (Fig. S12B–C), which were associated with mucosal protection and anti-inflammatory effects. Family-level clustering further revealed an enrichment of Muribaculaceae and Lactobacillaceae. These families are key taxa involved in polysaccharide degradation, short-chain fatty acid production, and epithelial barrier reinforcement. In contrast, pathogenic families, including Clostridiaceae and Peptostreptococcaceae, were significantly reduced (Fig. 5H–J, S12D-E).

Correlation analysis revealed that the shifts in microbial composition were closely associated with host physiology. Beneficial taxa such as Muribaculaceae and *Lactobacillus* showed negative correlations with pro-inflammatory markers, underscoring their role of mitigating mucosal inflammation (Fig. 5K). These findings indicated that BIO@PCS and BIO treatments alleviated colitis not only by restoring microbial diversity but also by selectively enriching taxa that reinforced epithelial integrity, antagonize pathogenic bacteria, and modulate immune responses. By reestablishing microbial homeostasis and strengthening the intestinal barrier, biofilm-state *L. paracasei* SB27 demonstrated strong therapeutic potential for the prevention and treatment of IBD and related inflammatory disorders.

## 4 Conclusion

Orally administered biofilm-state *L. paracasei* SB27 showed superior GI resistance, adhesion and colonization to intestinal epithelial cells compared to its planktonic counterpart. The dual-coating strategy with chitosan and PDA successfully enabled the targeted delivery and controlled release of biofilm-state *L. paracasei* SB27 to ulcerative sites. This approach facilitated the formation of a protective bacterial barrier in the inflamed region, functioning like a biological band-aid to alleviate colonic inflammation. By reinforcing intestinal defenses through mechanical protection (tight junction proteins), chemical protection (Mucin-2), immune regulation (inflammatory cytokines), and biological modulation (gut microbiota), this barrier effectively promoted the restoration of intestinal homeostasis. Integrating the inherent biological advantages of biofilm-state probiotics with a targeted delivery system, this strategy not only compensates for the low intestinal tolerance and poor targeting of traditional planktonic probiotics, but also offers a more effective and promising alternative to conventional pharmacological IBD therapies. Beyond this application, biofilms represent versatile biomaterials with broad potential in both therapeutic and biotechnological fields. Their structural stability, biocompatibility, and functional diversity support the development of biofilm-based hydrogels and bioactive components, such as exopolysaccharides and bacteriocins, for wound healing and anti-infective coatings.

The BIO@PCS platform demonstrates significant potential for clinical translation. Its simple and scalable preparation process allows for large-scale production with consistent quality. Both PDA and chitosan are biocompatible materials with safety profiles in biomedical applications. Additionally, BIO@PCS can be integrated with existing IBD treatments to enhance therapeutic effects. These attributes highlight the clinical feasibility of BIO@PCS as a safe, adaptable, and viable probiotic delivery system. However, the molecular mechanisms underlying the enhanced adhesion and function of biofilm-state probiotics remain to be fully elucidated. Future studies should focus on exploring the specific cellular targets and signaling pathways by which biofilm-state *L. paracasei* SB27 exerts its “band-aid” effect.

## CRediT authorship contribution statement

**Yinxue Liu:** Writing – original draft, Visualization, Validation, Project administration, Methodology, Investigation, Formal analysis, Data curation, Conceptualization. **Yisuo Liu:** Validation, Software, Investigation, Formal analysis. **Lu Jiang:** Validation, Methodology, Formal analysis. **Tongjie Liu:** Visualization, Investigation. **Zhe Zhang:** Writing – review & editing, Resources. **Pimin Gong:** Writing – review & editing, Supervision, Resources, Conceptualization. **Huaxi Yi:** Writing – review & editing, Visualization, Supervision, Project administration, Investigation, Funding acquisition, Conceptualization.

## Declaration of competing interest

The authors declare no conflict of interest.

## Declaration of competing interest

The authors declare that they have no known competing financial interests or personal relationships that could have appeared to influence the work reported in this paper.

## Data Availability

Data will be made available on request.
