# Supplementary material for: Polydopamine–chitosan coated biofilm-state Lacticaseibacillus paracasei SB27 as a living band-aid for targeted colitis therapy
Source: Mater Today Bio. 2025 Oct 24;35:102460. doi: 10.1016/j.mtbio.2025.102460 (PMC12596216; doi:10.1016/j.mtbio.2025.102460)
Supplement: Multimedia component 1 [file mmc1.docx]

**Polydopamine–chitosan coated biofilm-state *Lacticaseibacillus paracasei* SB27 as a living band-aid for targeted colitis therapy**

**Yinxue Liu^a^, Yisuo Liu^a^, Lu Jiang^a^, Tongjie Liu^a^, Zhe Zhang^a^, Pimin Gong^a^*, Huaxi Yi^a^***

**
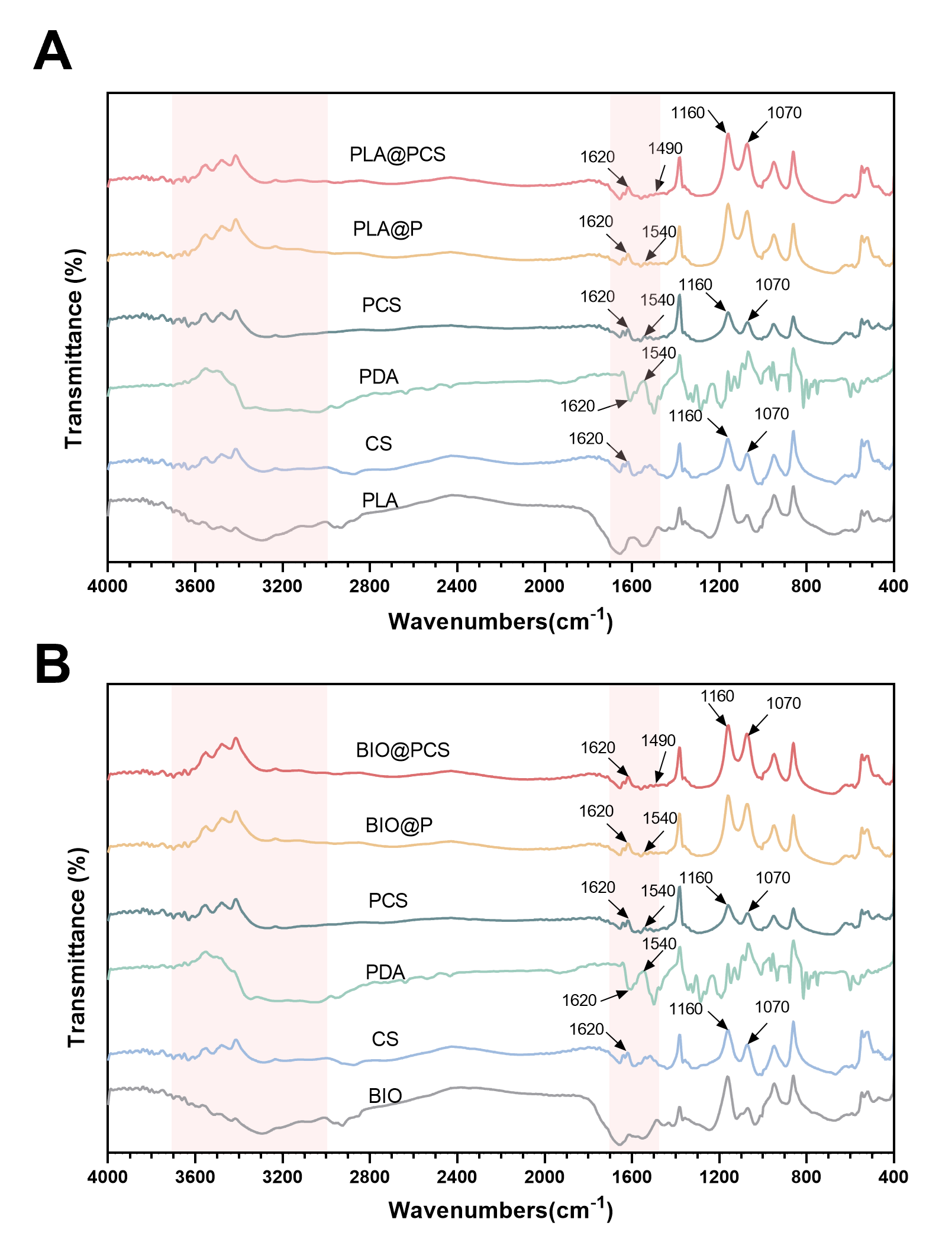
**

**Fig. S1.** FTIR characterization of PDA/CS-based coatings on planktonic and biofilm cells. **A**) Planktonic cells. **B**) Biofilm cells.

**
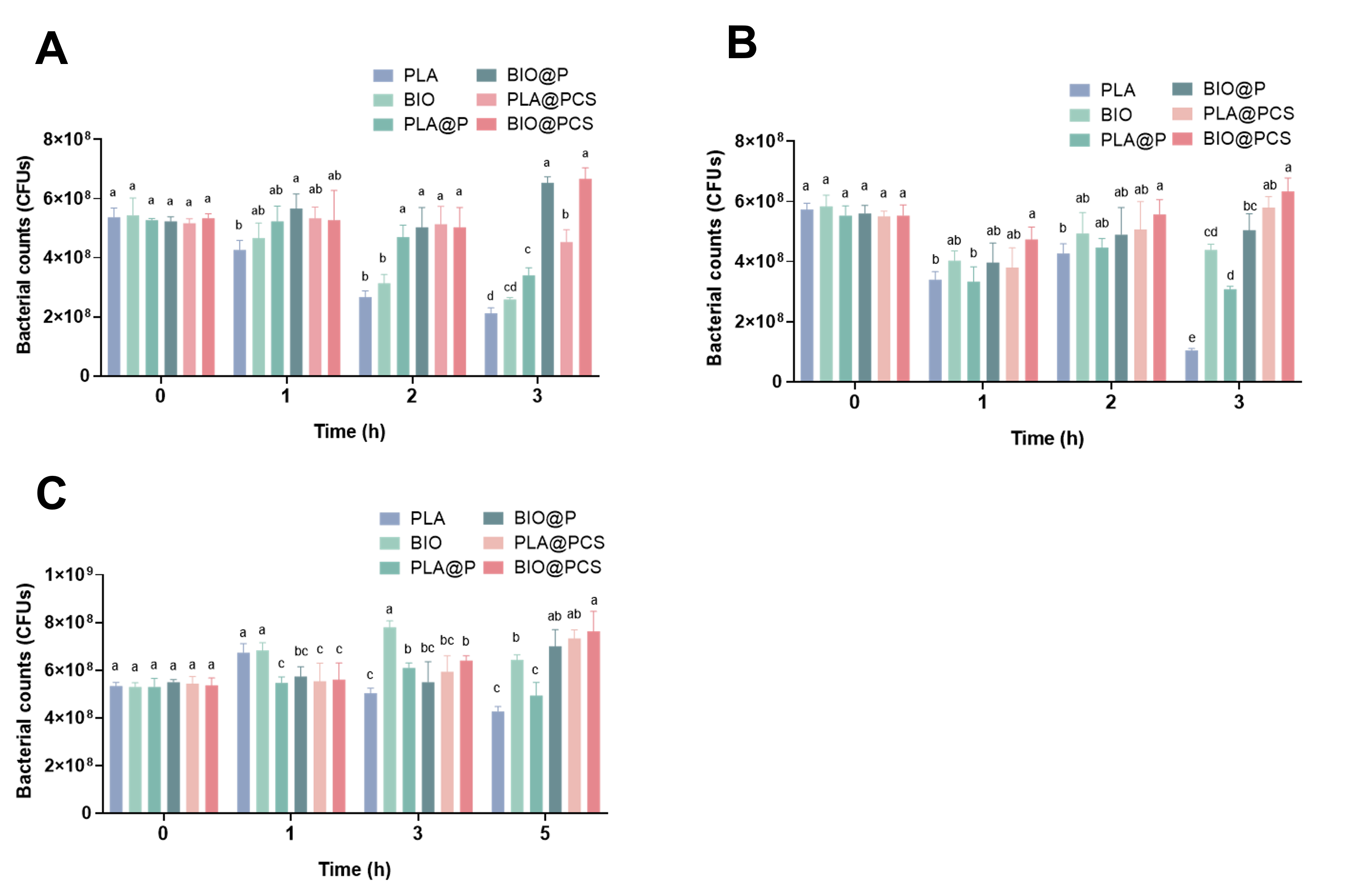
**

**Fig. S2.** Assessment of bile salt and GI fluid tolerance. **A**) Bile salt tolerance. **B**) Simulated gastric fluid tolerance. **C**) Simulated intestinal fluid tolerance. Data are presented as means ± SD (n = 3). Different lowercase letters indicated significant differences (*p* < 0.05).

**
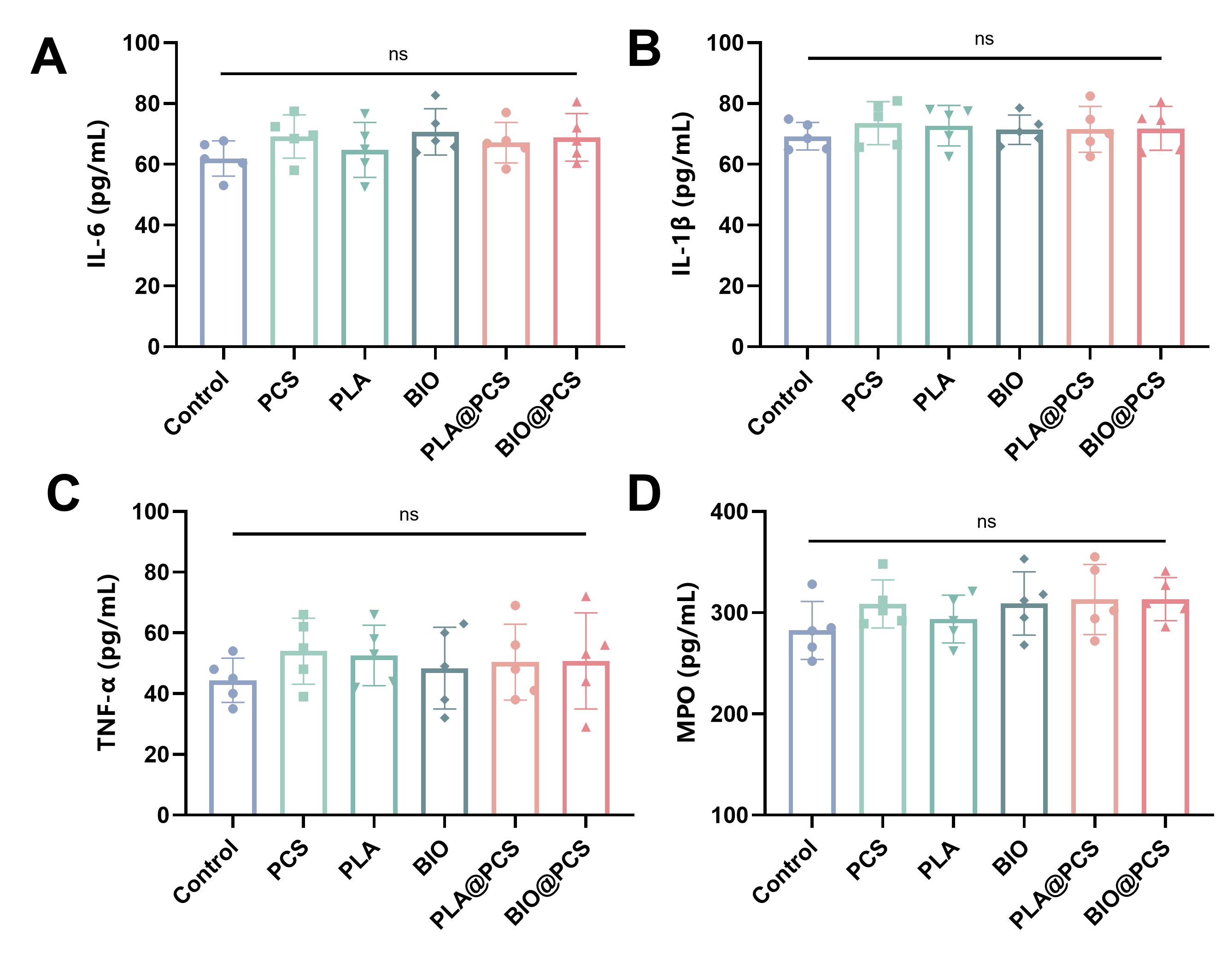
**

**Fig. S3.** Cytokine assays including **A)** IL-6, **B)** IL-1β, **C**) TNF-α, **D)** MPO in serum measured by commercially available ELISA kits. Data are presented as means ± SD (n = 5). Significance was assessed by one-way analysis of variance (ANOVA) with Tukey post hoc test, giving *p*-values, ns, not significant.

**
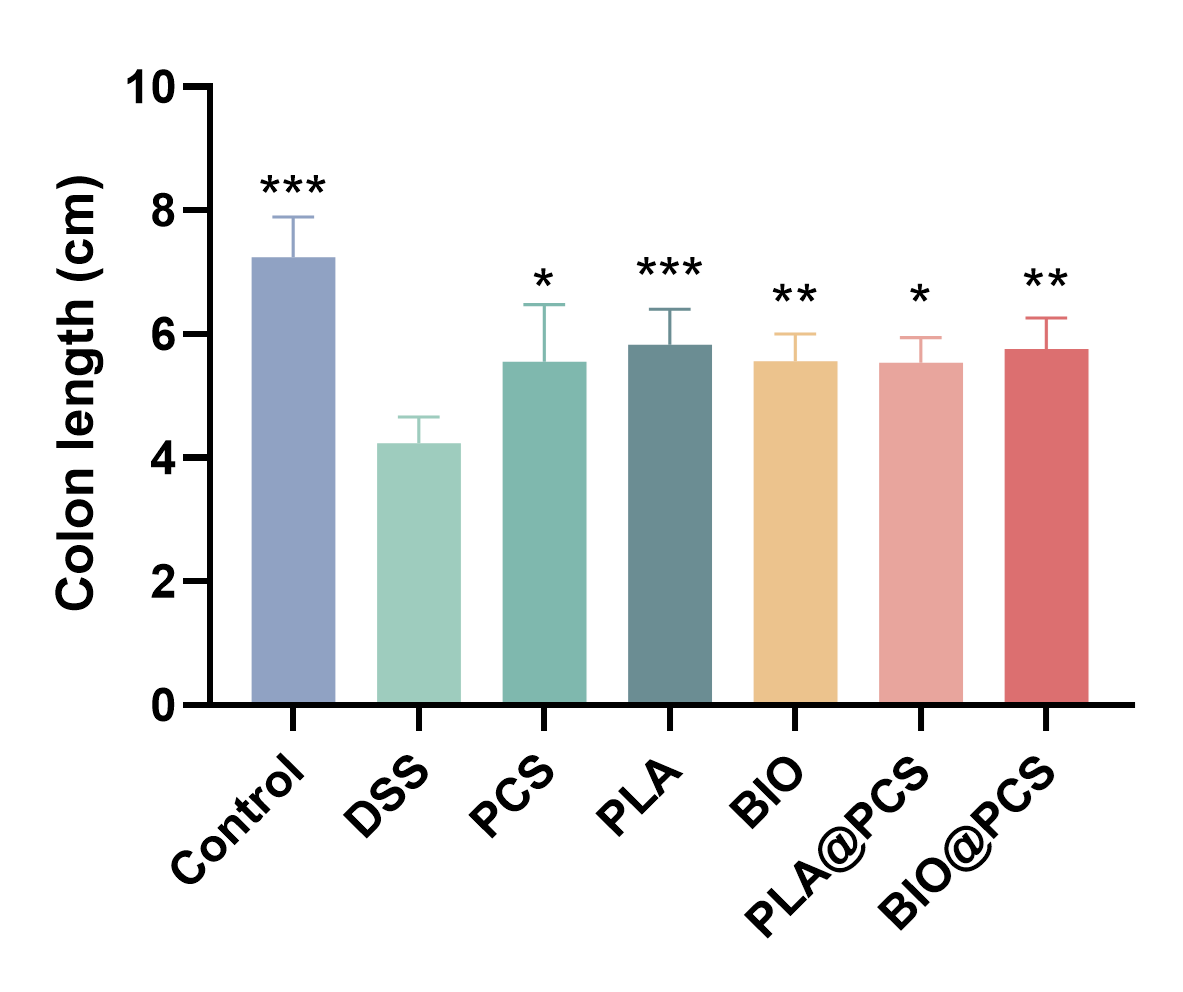
**

**Fig. S4.** Corresponding quantified lengths of colons harvested from mice of different groups on day 22. Data are presented as means ± SD (n = 6). Significance was assessed by one-way analysis of variance (ANOVA) with Tukey post hoc test, giving *p*-values, **p* < 0.05, ***p* < 0.01, ****p* < 0.001.


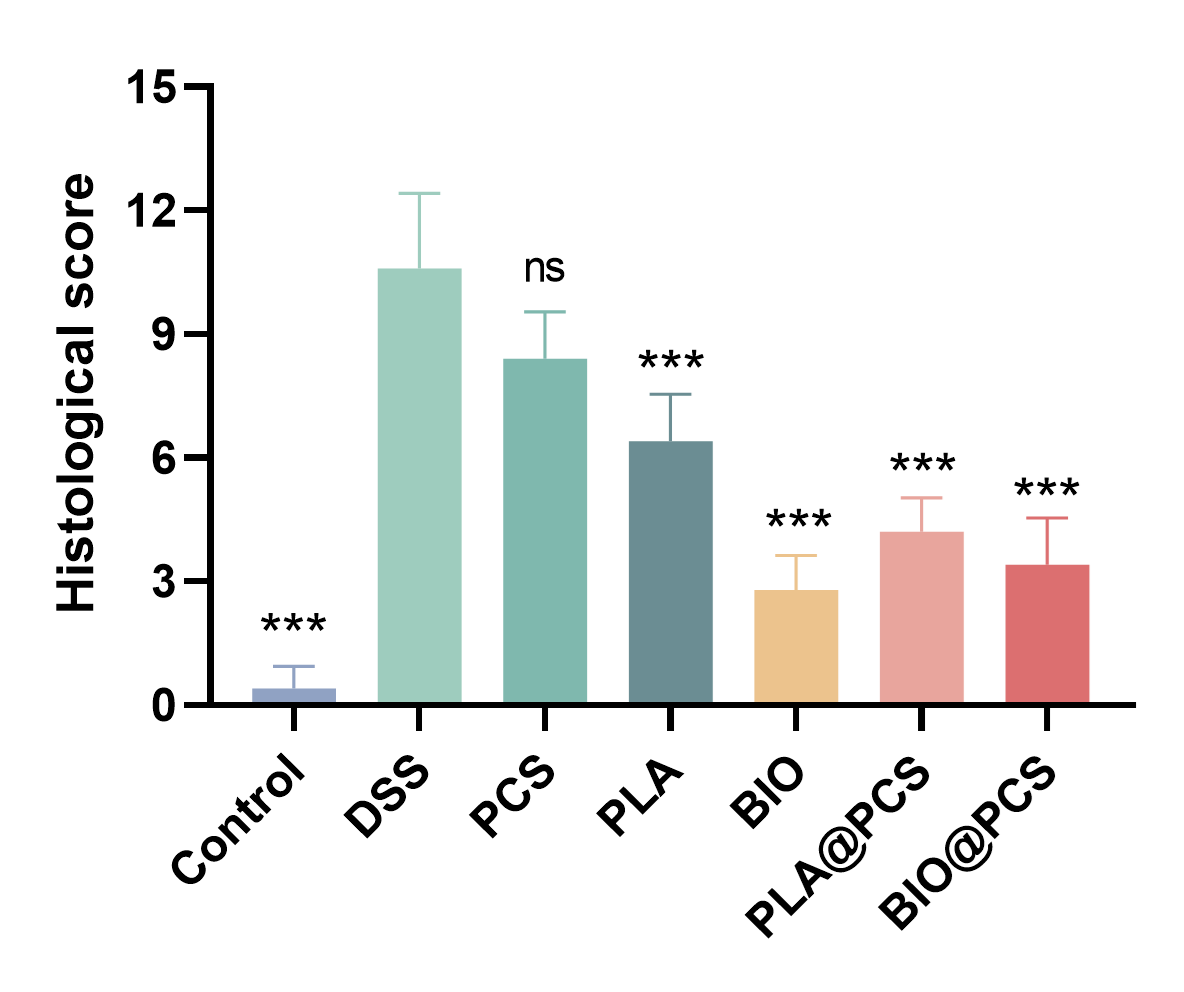


**Fig. S5.** Colonic histological damage scores. Data are presented as means ± SD (n = 6). Significance was assessed by one-way analysis of variance (ANOVA) with Tukey post hoc test, giving *p*-values, ****p* < 0.001; ns, not significant.

**
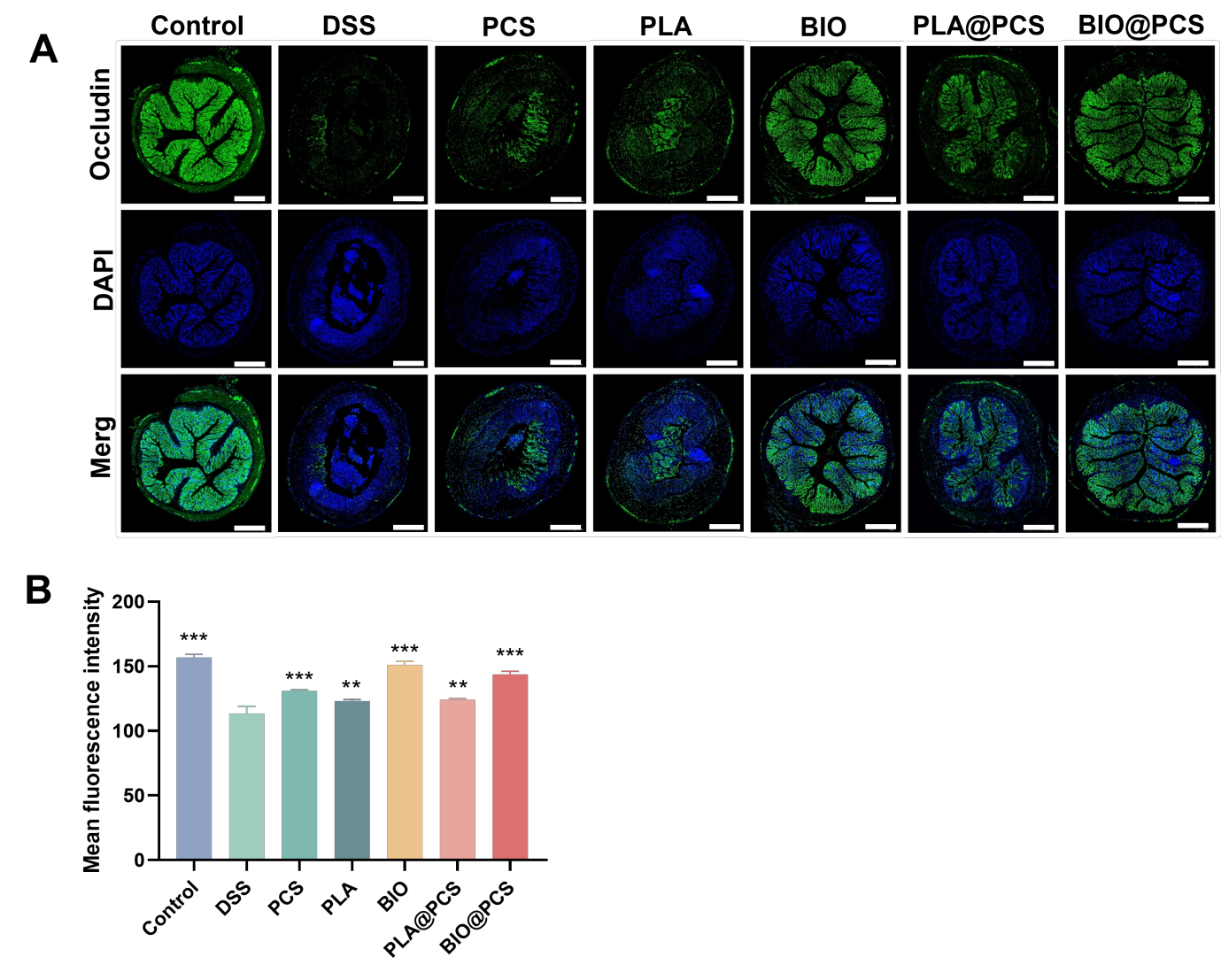
**

**Fig. S6.** The expression of tight junction protein Occludin. **A**) Representative immunofluorescence staining images. Scale bars: 400 μm. **B**) Mean fluorescence intensity of colon sections. All fluorescence images were acquired with consistent exposure and laser intensity settings, and consistently processed (brightness/contrast adjustment and background subtraction) for comparability. Data are presented as means ± SD (n = 3). Significance was assessed by one-way analysis of variance (ANOVA) with Tukey post hoc test, giving *p*-values, ***p* < 0.01, ****p* < 0.001.

**
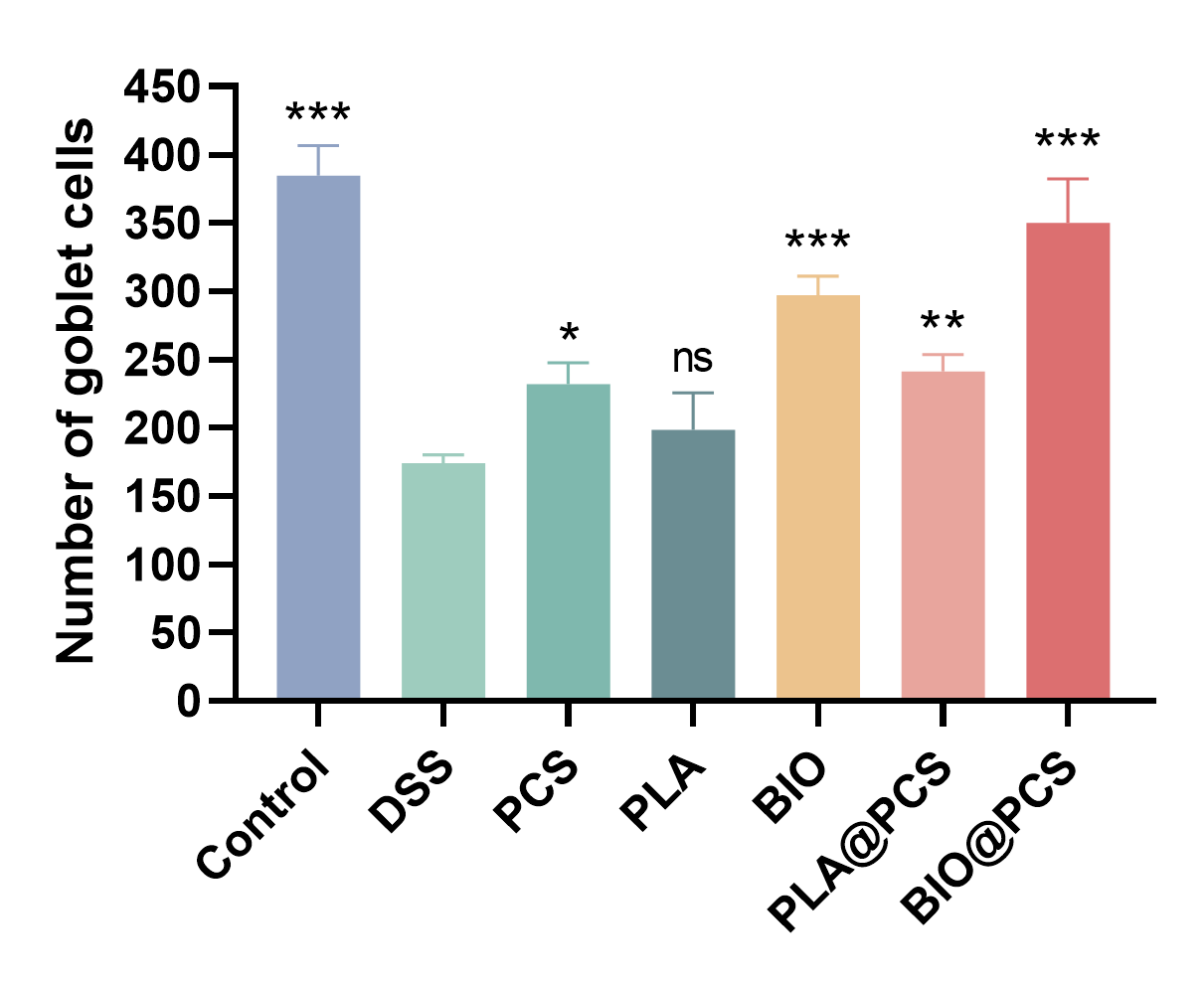
**

**Fig. S7.** The number of goblet cells in colon tissue was stained by AB-PAS. Data are presented as means ± SD (n = 6). Significance was assessed by one-way analysis of variance (ANOVA) with Tukey post hoc test, giving *p*-values, **p* < 0.05, ***p* < 0.01, ****p* < 0.001; ns, not significant.


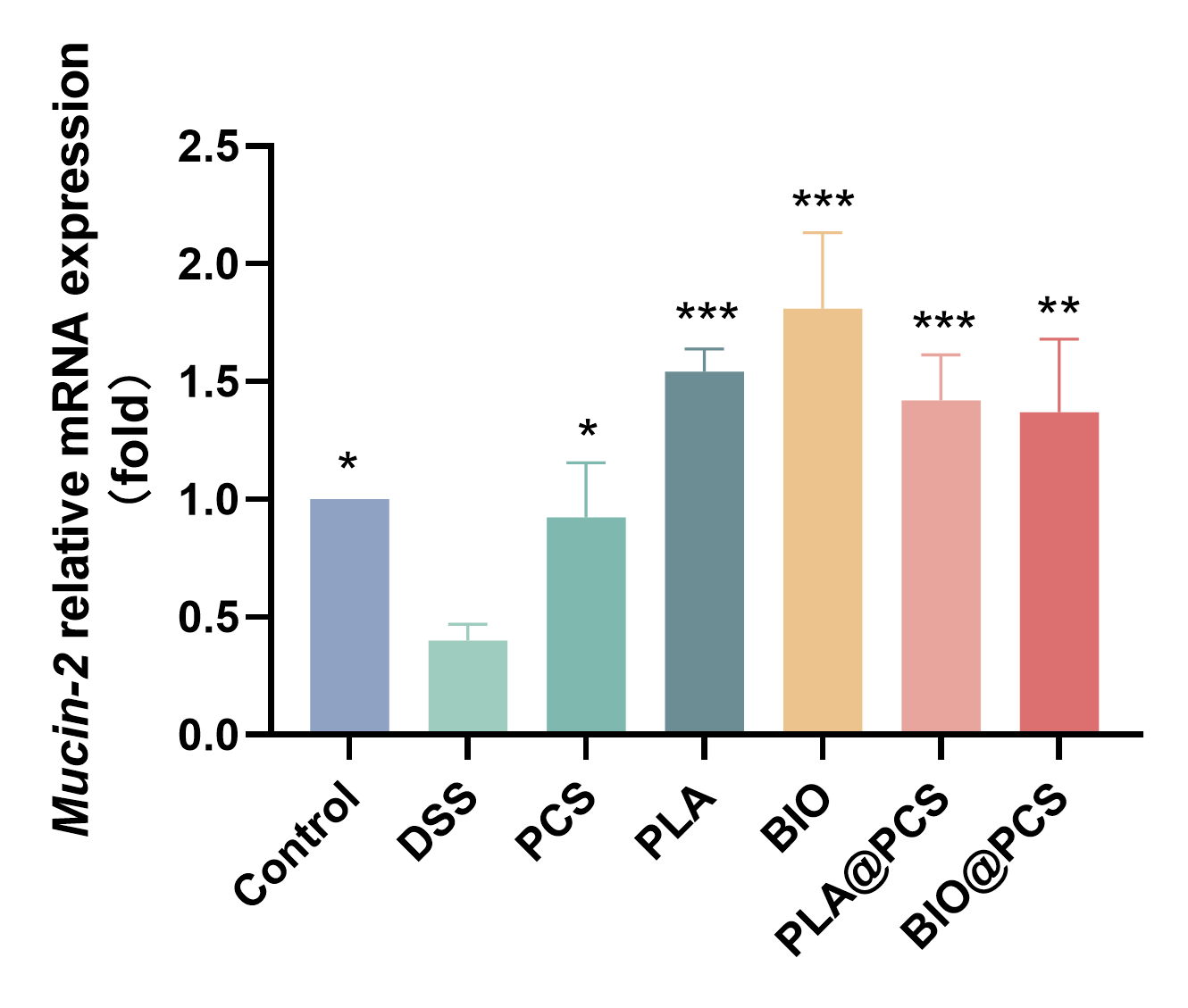


**Fig. S8.** Expression of *Mucin-2* gene in mouse colon. Data are presented as means ± SD (n = 3). Significance was assessed by one-way analysis of variance (ANOVA) with Tukey post hoc test, giving *p*-values, **p* < 0.05, ***p* < 0.01, ****p* < 0.001.

**
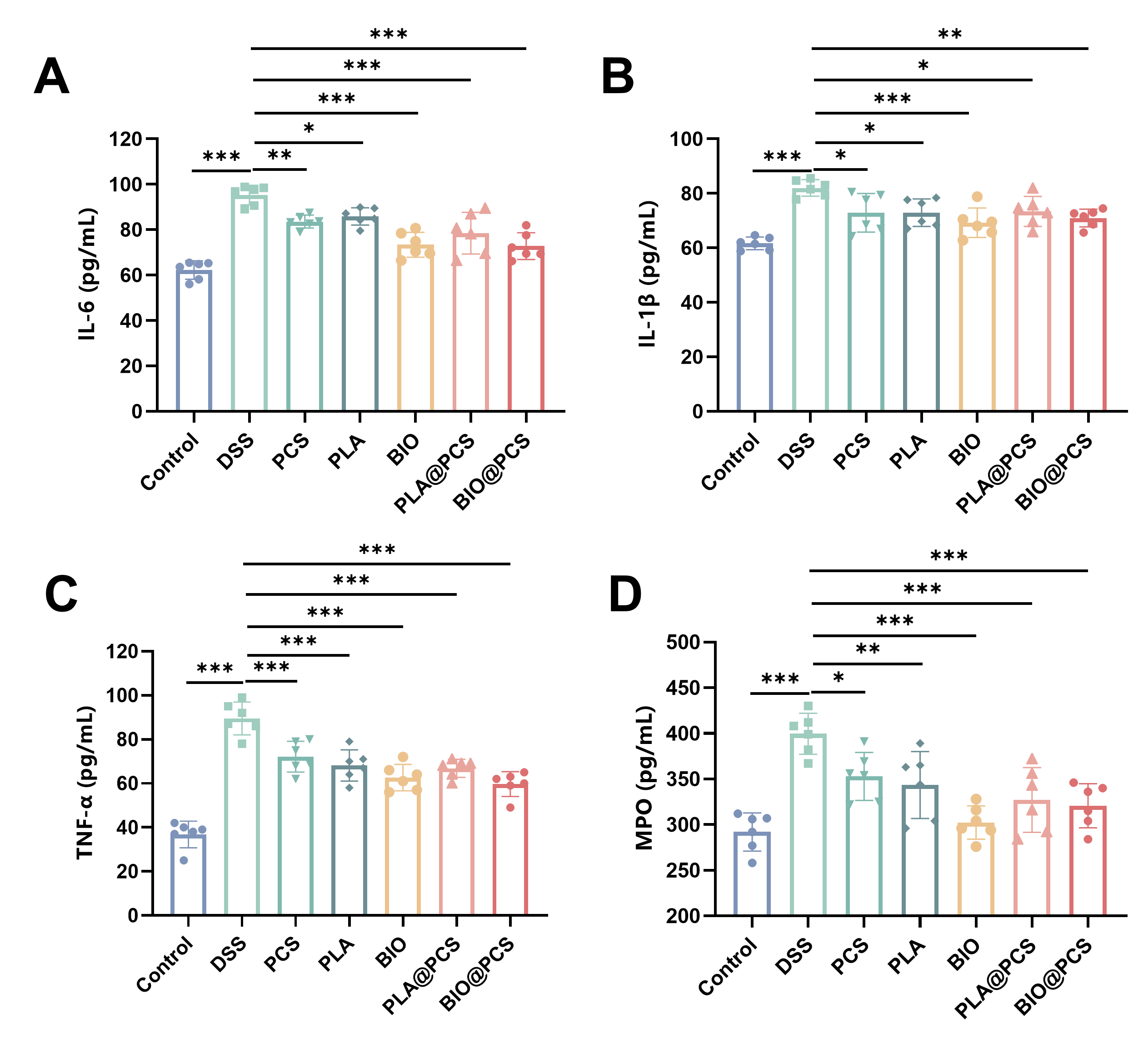
**

**Fig. S9.** Cytokine assays including **A)** IL-6, **B)** IL-1β, **C**) TNF-α, **D)** MPO in serum measured by commercially available ELISA kits. Data are presented as means ± SD (n = 6). Significance was assessed by one-way analysis of variance (ANOVA) with Tukey post hoc test, giving *p*-values, **p* < 0.05, ***p* < 0.01, ****p* < 0.001.

**
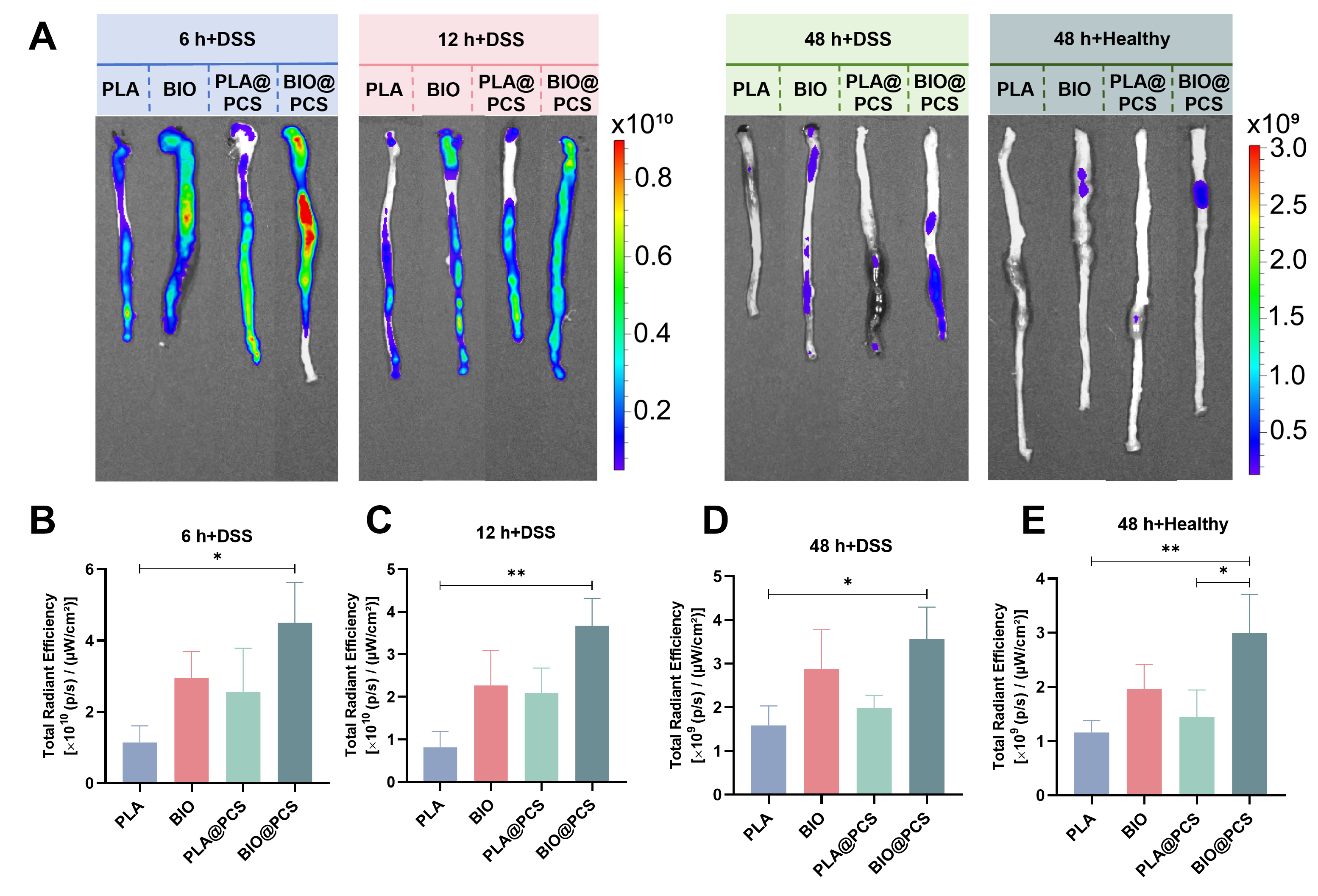
**

**Fig. S10.** Targeted accumulation of BIO@PCS in the inflamed murine colon. **A**) Fluorescence images of the mouse colon at different times after oral administration of PLA, BIO, PLA@PCS and BIO@PCS. **B - D)** Fluorescence intensity in the colon of DSS-induced mice at 6 h **B**), 12 h **C**), and 48 h **D**) post-gavage. **E**) Fluorescence intensity in the colon of healthy mice at 48 h post-gavage. All fluorescence images were acquired with consistent exposure and laser intensity settings, and uniformly processed (brightness/contrast adjustment and background subtraction) for comparability. The color scale was optimized to clearly visualize biodistribution differences across time points: higher ranges were used for the 6 h + DSS and 12 h + DSS groups, and lower ranges for the 48 h + DSS and 48 h + Healthy groups. Data are presented as means ± SD (n = 3). Significance was assessed by one-way analysis of variance (ANOVA) with Tukey post hoc test, giving *p*-values, **p* < 0.05, ***p* < 0.01.

**
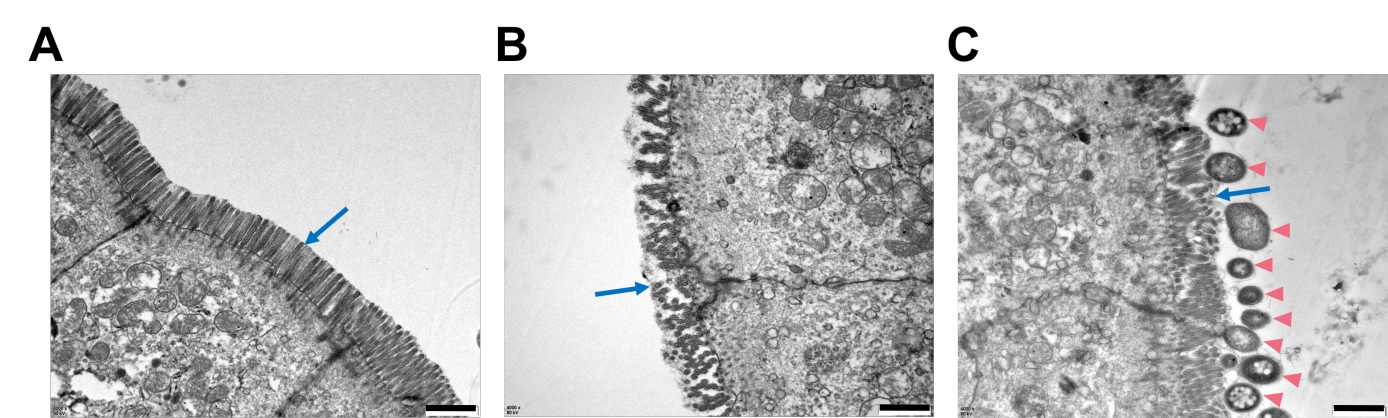
**

**Fig. S11.** Representative TEM images of murine colonic tissue. **A**) Healthy colon. **B**) Inflamed colon. **C**) Targeted colonization of BIO@PCS in inflamed colon at 12 h post oral administration. Scale bars: 1 μm. Blue arrows indicate the intestinal mucosal layer, and pink arrows indicate colonized BIO@PCS.

**
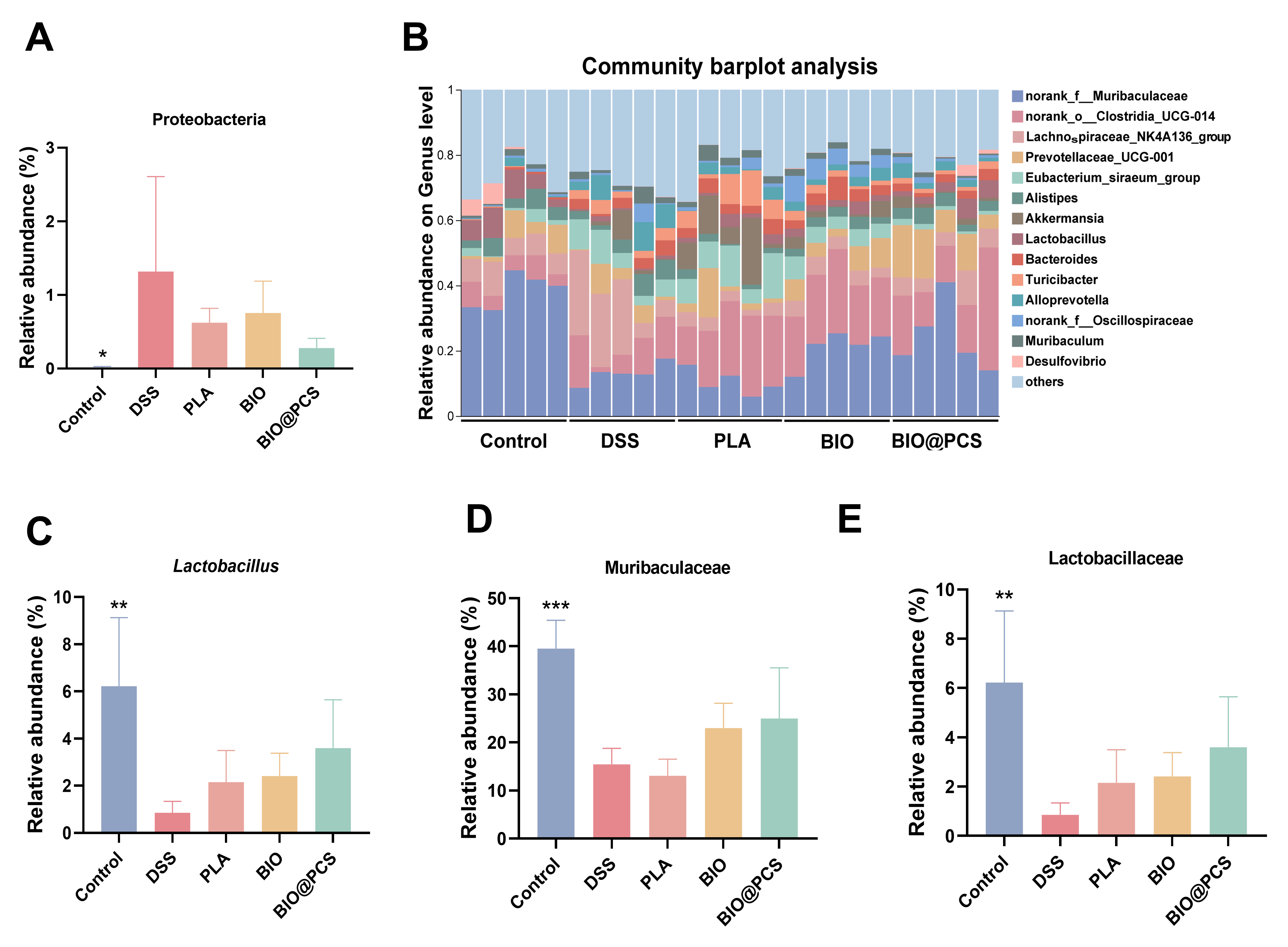
**

**Fig. S12.** Changes in the gut microbiota of mice. **A**) Relative abundance of Proteobacteria. **B)** Histogram of the relative abundances of different species on genus level. **C**) Relative abundance of *Lactobacillus.* **D**) Relative abundance of Muribaculaceae. **E**) Relative abundance of Lactobacillaceae. Data are presented as means ± SD (n = 5). Significance was assessed by one-way analysis of variance (ANOVA) with Tukey post hoc test, giving *p*-values, **p* < 0.05, ***p* < 0.01, ****p* < 0.001.
